# The BAF chromatin remodeling complex licenses planarian stem cells access to ectodermal and mesodermal cell fates

**DOI:** 10.1186/s12915-023-01730-y

**Published:** 2023-10-20

**Authors:** Mallory Wiggans, Shu Jun Zhu, Alyssa M. Molinaro, Bret J. Pearson

**Affiliations:** 1 The Hospital for Sick Children, Program in Developmental and Stem Cell Biology, Toronto, ON M5G0A4 Canada; 2 Department of Molecular Genetics, University of Toronto, Toronto, ON M5S1A8 Canada; 3 Present address: Oregon Health & Science University, Portland, OR 97239 USA

**Keywords:** Adults stem cells, Stemness, Planarians, Chromatin remodeling, BAF complex, Brg1, smarcc2

## Abstract

**Background:**

The flatworm planarian, *Schmidtea mediterranea*, has a large population of adult stem cells (ASCs) that replace any cell type during tissue turnover or regeneration. How planarian ASCs (called neoblasts) manage self-renewal with the ability to produce daughter cells of different cell lineages (multipotency) is not well understood. Chromatin remodeling complexes ultimately control access to DNA regions of chromosomes and together with specific transcription factors determine whether a gene is transcribed in a given cell type. Previous work in planarians determined that RNAi of core components of the BAF chromatin remodeling complex, *brg1* and *smarcc2*, caused increased ASCs and failed regeneration, but how these cellular defects arise at the level of gene regulation in neoblasts is unknown.

**Results:**

Here, we perform ATAC and RNA sequencing on purified neoblasts, deficient for the BAF complex subunits *brg-1* and *smarcc2*. The data demonstrate that the BAF complex promotes chromatin accessibility and facilitates transcription at target loci, as in other systems. Interestingly, we find that the BAF complex enables access to genes known to be required for the generation of mesoderm- and ectoderm-derived lineages, including muscle, parenchymal cathepsin, neural, and epithelial lineages. BAF complex knockdowns result in disrupted differentiation into these cell lineages and functional consequences on planarian regeneration and tissue turnover. Notably, we did not detect a role for the BAF complex in neoblasts making endodermal lineages.

**Conclusions:**

Our study provides functional insights into how the BAF complex contributes to cell fate decisions in planarian ASCs in vivo.

**Supplementary Information:**

The online version contains supplementary material available at 10.1186/s12915-023-01730-y.

## Background

Adult stem cells (ASCs) self-renew throughout the life of an organism and produce differentiated daughter cells of multiple cell types (multipotency). A major question in stem cell biology is how ASCs remain undifferentiated while still having access to the suites of gene expression that can drive multiple cellular outputs. The routes to different programs of differentiation are ultimately controlled at the level of chromatin, and chromatin remodeling complexes are key players in determining access to genomic loci as well as overall chromatin compaction that occurs during differentiation [1–3]. Understanding how ASCs interpret and integrate signals to regulate chromatin, gene expression, and ultimately cell fate choice is critical to explaining intricate developmental processes like tissue homeostasis and regeneration.

ASCs are known to utilize chromatin-remodeling complexes to balance self-renewal and differentiation programs [3–7]. Among many examples and complexes, the BRG/BRM-Associated Factor (BAF) chromatin remodeling complex is evolutionarily conserved from yeast (SWI/SNF) to mammals [8–11]. In animals, BAF tends to open chromatin to facilitate gene transcription [12–18]; however, it can also repress loci [16, 19, 20], as well as act variably at enhancers [21]. Mammalian BAF (mBAF) complexes share a common core consisting of a ATP-hydrolyzing catalytic subunit (BRG1 or BRM; encoded by *smarca4* and *smarca2,* respectively) with accessory subunits BAF47, BAF155, BAF170 (encoded by*, smarcb1*, *smarcc1*, and *smarcc2*, respectively), together of which constitute the minimum requirement for nucleosome remodeling in vitro [13]. mBAF contains up to 15 subunits [8, 22], many of which are encoded by gene families where different paralogs can be incorporated into the complex in a mutually exclusive manner so that the complex has cell-type-specific functions. BAF complex compositions can be unique to specific tissues and processes such as neural development, neural turnover [23–27], and heart development [28–30]. For instance, the BAF complex interacts with Pax6 to initiate a neurogenic program in murine adult neural progenitors [31]. Further, in murine cardiac development, the BAF complex directs expression of specific genes required for cardiac mesoderm development [29]. Reprogramming studies determined the BAF complex is also required to safeguard against dedifferentiation and transdifferentiation after lineage specification [32]. In total, the BAF complex has critical roles in controlling expression of key genes that initiate cell specification and differentiation of specific cell lineages during development and beyond.

In terms of stem cell biology, mammals utilize the embryonic stem cell BAF complex (esBAF) to ensure self-renewal and pluripotency. The esBAF exclusively contains BRG1 rather than BRM and interacts with key regulators of pluripotency such as OCT4, NANOG, and SOX2 [17, 20]. In addition to roles in stem cells and development, the BAF complex is a driver of multiple cancers where it is estimated that mutations in the BAF complex occur in >20% of all human cancers [33] and high expression of *brg1* correlates with poor overall survival in many cancer types [34]. Despite such critical roles in mammalian stem cell biology, development, and disease, it is not entirely clear how specific BAF complex subunits function in ASC biology, in part due to challenges of assaying perturbations of stem cell lineages in adults in vivo. However, understanding BAF functions in ASC biology can be aided by non-traditional systems such as the freshwater planarian, *Schmidtea mediterranea*, which has homologs for BRM and BRG as well as other mBAF subunits [35], and contains experimentally accessible ASCs.

Planarians are well-known for their ability to perform whole-body regeneration, which is dependent on their large population of ASCs—called neoblasts [36–40]. Neoblasts comprise 10–20% of all cells in the animal [37–40] and demonstrate self-renewal and pluripotency [41, 42]. Comparative studies have demonstrated that neoblasts share common stemness programs with mammals, suggesting conserved molecular programs exist in stem cells of diverse species [43–46]. In addition to regeneration, neoblasts are required for homeostatic cell turnover of all adult planarian tissues, and key cell fate regulators are expressed in neoblasts to drive differentiation programs for tissues including the CNS, gut, protonephridia, muscle, and pharynx [38, 47–53]. Further, planarians have tools that facilitate genomic studies such as a sequenced and assembled genome [54]; high-confidence gene predictions; transcriptomic data from purified neoblasts in WT and various RNAi conditions [44, 55, 56]; and multiple single-cell RNA-sequencing (scRNAseq) cell atlases [57–59]. Finally, RNAi can be performed in adult animals to achieve gene knockdown without concern for embryonic lethality [60, 61]. In total, these advantages facilitate the use of planarians to investigate the genetic pathways that contribute to neoblast function and cell fate decisions in vivo.

Previous studies have implicated multiple chromatin remodeling complexes in the regulation of neoblast functions (e.g., self-renewal, differentiation, proliferation) [42, 62–68]. Regarding the BAF complex, planarians have orthologs for all core mammalian BAF complex subunits [35]. Our lab and others have found that both core subunits, *brg1* and *smarcc2*, have enriched expression in neoblasts when compared to the rest of the animal [35, 46, 47, 55]. RNAi of *brg1* or *smarcc2* results in expansion of neoblasts and increased cell division, with a concomitant loss of epidermal progenitors [35, 46, 69]. Despite implication of the BAF complex in balancing stem cell function and differentiation, it remains unknown how it exerts its functions at the chromatin level to allow for differentiation in planarians, nor what lineages may be affected beyond the epithelium.

Here, we demonstrate that the BAF complex regulates chromatin accessibility at genomic loci of genes known to be required for differentiation down multiple lineages. Bulk RNA and ATAC sequencing from purified neoblasts in *control*, *brg1*, or *smarcc2* RNAi conditions, demonstrates reduced chromatin accessibility and transcription at genes associated with ectodermal and mesodermal lineages, while endodermal lineages were largely unaffected. Upon amputation, planarians are unable to regenerate positional control gene (PCG)-expressing muscle cells. During homeostasis, RNAi of the core subunits of the BAF complex results in the inability to specify epidermal progenitor cells and differentiate down the epidermal lineage. Finally, putative neural progenitor cells are reduced, and there is a reduction in the rate of neural turnover when BAF function is knocked down. Together, our study indicates that the planarian BAF complex is required for neoblasts to access specific differentiated lineages during both tissue turnover and during regeneration in order to balance self-renewal with differentiation.

## Results

### *brg1* and *smarcc2* are expressed widely across neoblasts and progenitors

**Fig. 1:**
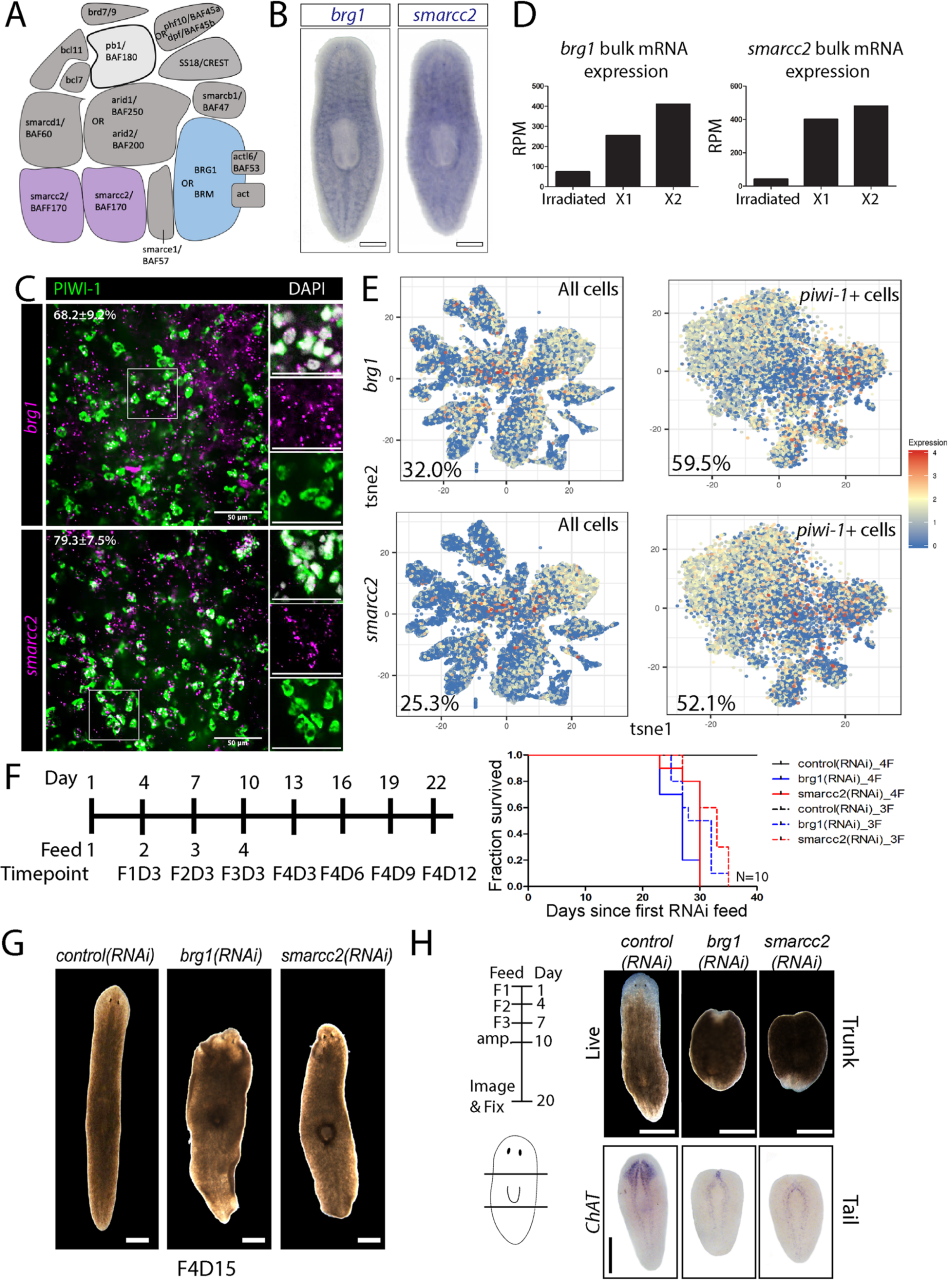
*brg1* and *smarcc2* are expressed in stem cells and are required for tissue turnover and regeneration. **A** Schematic of putative planarian BAF complex based on homology with human BAF. Two protein products of *smarcc2* (BAF170) are predicted to be incorporated into a functional complex. The core components *smarcc2* and *brg1* are colored. The protein product of *pb1* (BAF180) is exclusively incorporated into polybromo BAF (pBAF) rather than canonical BAF (light gray). Gene and protein names are included on diagram unless the names are the same. Adapted from [70]. **B** WISH of BAF core components in wildtype planarians imaged dorsally. Scale bars, 200µm. **C** FISH showing *brg1* or *smarcc2* expression with α-PIWI-1 (neoblasts and post-mitotic immediate progenitors). Quantifications are mean $\pm$ 1 S.D., *n* >8 (each “*n*” is a representative region from 1 planarian). Scale bars, 50µm. **D** Planarian *brg1* and *smarcc2* expression in FACS-isolated X1s (neoblasts), X2s (G0/G1 neoblasts and immediate progeny), and irradiated (differentiated tissues) cell fractions determined using bulk RNAseq [44, 48]. RPM, reads per million mapped reads. **E** tSNE representation of single-cell RNA sequencing of whole worm (left) and *piwi-1*+ cells (neoblasts) only (right). Plots are colored according to gene expression (red, high; blue, low) for *brg1* (top panels) or *smarcc2* (bottom panels). Data are from [58]. **F** RNAi paradigm used in experiments. Days from initiation of experiment, feed number, and timepoints are indicated (e.g., F4D3 denotes four feeds, day 3 after the fourth feed). Survival curves demonstrate 100% lethality with either three or four feeds in both *brg1* and *smarcc2* RNAi. **G** Live images of *control(RNAi)*, *brg1(RNAi)*, and *smarcc2(RNAi)* planarians. Scale bars, 200µm. **H** Live images and WISH for the neural marker *ChAT* of *control(RNAi)*, *brg1(RNAi)*, or *smarcc2(RNAi)* planarians during regeneration. Experimental timeline and amputation sites are depicted to the left of the images (*n* >10). Scale bars, 200µm

Planarians possess orthologs of all essential subunits present in mBAF complexes (Fig. 1A; Additional file 1) and RNAi of *brg1* or *smarcc2* results in neoblast defects and disruption of the epithelial lineage [35, 46]. In order to predict where *brg1* and *smarcc2* might function, we first examined their expression. By whole-mount in situ hybridization (WISH), *brg1* and *smarcc2* were detected in neoblast-like patterns, demonstrated by strong staining in the parenchyma between gut branches and in the tail where neoblasts reside (Fig. 1B). Immunostaining with α-PIWI-1 (a kind gift of Dr. Jochen Rink), which labels neoblasts and *piwi-1*- immediate post-mitotic progenitor cell types, combined with fluorescent in situ hybridization (FISH) of *brg1* or *smarcc2* demonstrated colocalization of the subunit expression in neoblasts and immediate progenitors in vivo (Fig. 1C). Similarly, published bulk RNA sequencing of flow cytometry-purified X1 (neoblasts), X2 (G0/G1 neoblasts and immediate post-mitotic progeny), and Xins (differentiated cells) demonstrated that *brg1* and *smarcc2* were enriched in X1s and X2s over differentiated cell types (Fig. 1D) [43, 47]. Finally, published scRNAseq cell atlas data indicated *brg1* and *smarcc2* were detected in 32.0 and 25.3% of all cells, and 59.5 and 52.1% of all *piwi-1*+ neoblasts cells respectively (Fig. 1E) [57]. Most of the other identified homologs for the BAF complex subunits displayed similar expression by WISH, scRNAseq, and bulk RNA sequencing as *brg1* and *smarcc2* with the exception of the homologs for *brm-1*, *bcl11-1* and *bcl11-2* (Additional file 2) [57]. In total, we find that BAF subunits are expressed widely across neoblasts and their immediate progeny.

### brg1 and smarcc2 are required for tissue turnover and regeneration

In order to test the function of *brg1* and *smarcc2*, planarians were administered RNAi-food targeting either *brg1* or *smarcc2*, which resulted in 100% animal lethality with either three or four doses, consistent with previous studies (Fig. 1F) [35, 44, 46, 69]. A four-feed RNAi regimen was implemented for all subsequent experiments unless noted otherwise. Uninjured *brg1* or *smarcc2* RNAi animals displayed morphological phenotypes characteristic of neoblast dysfunction including dorsal lesions, head regression, ventral curling, and eventual lysis (Fig. 1G) [61]. In addition to cellular turnover, neoblasts are also required for tissue regeneration. Similar to the stem -cell defective phenotype during tissue homeostasis in *brg1* or *smarcc2* RNAi animals, regenerating animals showed stem cell defects, resulting in loss of all regenerative ability (Fig. 1H). Previous work demonstrated that RNAi of homologs of other subunits of the BAF complex, including *arid1*, *arid2-1*, and *smarcb1* also results these characteristic stem cell defective morphological phenotypes and impaired regeneration in planarians [35]. In total, the BAF complex was required for stem cell function; however, these morphological phenotypes could result from very different cellular perturbations. For example, stem cell defective phenotypes can arise from loss of stem cells, arrest of stem cells, or inability to produce differentiated progeny. We next tested between these possibilities.

### brg1 and smarcc2 are required for stem cells to differentiate

**Fig. 2:**
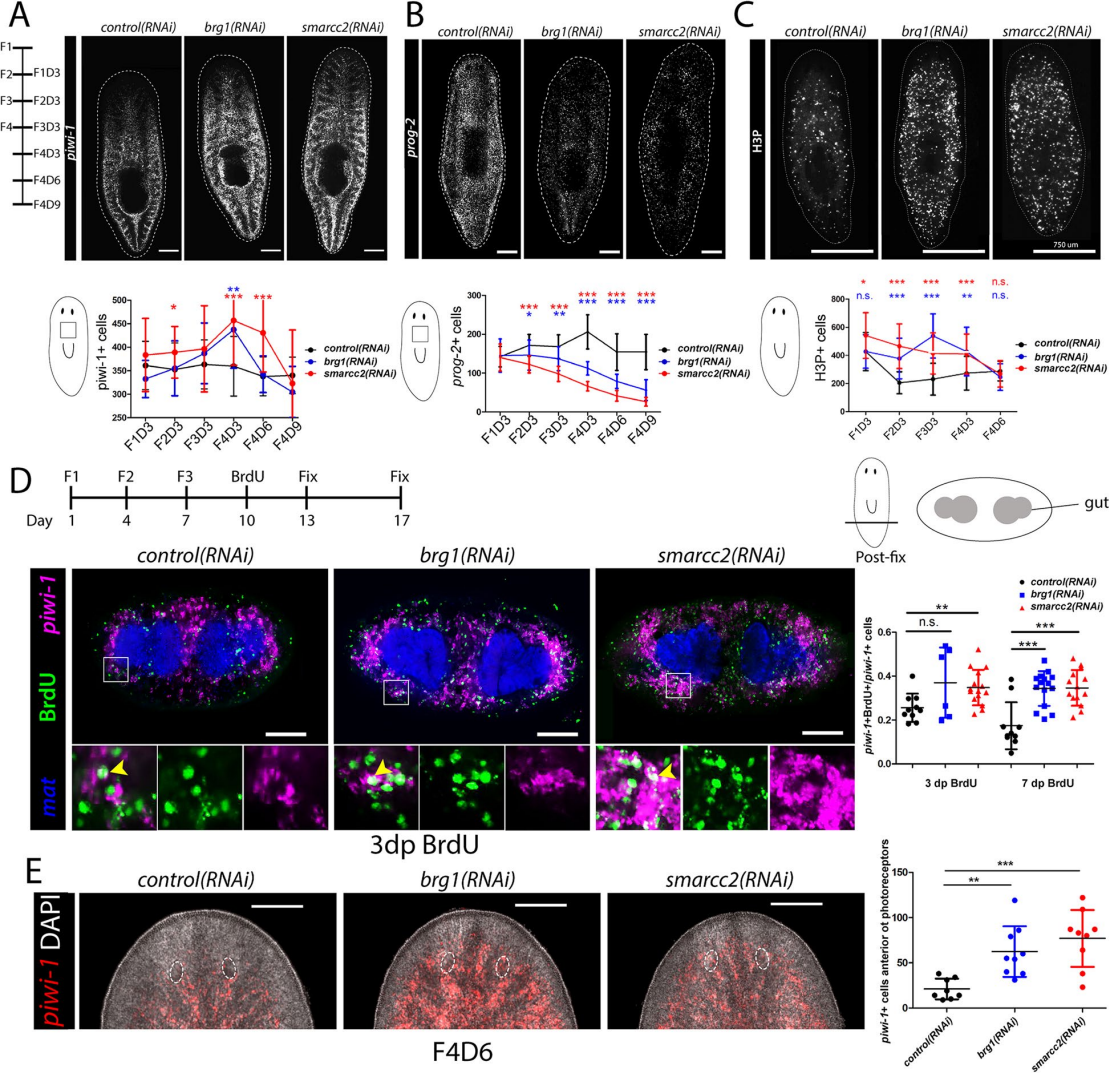
The BAF complex regulates neoblasts and is required for differentiation. **A** Representative maximum projections of FISH for *piwi-1* during RNAi at F4D3. *piwi-1*+ cells were analyzed according to the time course to left of images. Quantifications of number of *piwi-1+* cells in boxed region indicated on diagram below image are shown in graph below the image (*n* >18). Scale bars, 200μm. **B** Representative maximum projections of FISH for epidermal progenitor marker *prog-2* during RNAi at F4D3. *prog-2*+ cells were analyzed according to the time course in (**A**). Quantification of the number of *prog-2+* cells in boxed region indicated on diagram below image is shown in the graph below the image (*n* >18). Scale bars, 200μm. **C** Representative whole-animal immunostaining for H3P during RNAi at F4D3. Total H3P^+^ cells per worm area were counted at timepoints indicated in (**A**) and are quantified in the graph below images (*n* >18). Scale bars, 750µm. **D** Immunostaining of BrdU with *piwi-1* and *mat* dFISH for planarians subjected to RNAi followed by a BrdU pulse and chase period. An experimental timeline is indicated above images. Stained worms were cross-sectioned post-pharyngeally. Quantifications of the number of *piwi-1*+/BrdU+ to total *piwi-1*+ are shown in graph to the right. Scale bars, 100µm. *mat* is displayed for orientation to the gut. dp is “days post”. **E** Representative maximum projection of FISH for *piwi-1* in BAF RNAi intact heads. *piwi-1*+ cells anterior to the photoreceptors (location indicated by dashed circles) are quantified in the graph to the right. Scale bars, 100µm. Quantifications are mean $\pm$ 1 S.D Significance levels in plots: *p<0.05, **p<0.01, ***p<0.001, each *n* is a representative region from 1 planarian

As mentioned, *piwi-1*+ cells appear increased and *prog-2+* epithelial progenitor cells decreased in BAF RNAi conditions [35, 44, 46]. Furthermore, RNAi to homologs of other BAF subunits, *arid1* and *smarcb1*, also appear to have increased *piwi-1+* cells and reduced *prog-2+* cells, suggesting other mBAF complex components contribute to BAF function in planarians [35]. Performing time course analyses of cellular dynamics during RNAi phenotypes is a powerful method to determine how stem cell function is disrupted. To this end, we assayed neoblasts, early division progeny of the epithelial lineage, cell division, and cell death in whole animals every 3 days following *control*, *brg1*, or *smarcc2* RNAi (Fig. 2A–C). We observed that RNAi of either *brg1* or *smarcc2* had virtually identical phenotypes and that the stem cell compartment was expanded at the expense of *prog-2*+ early epithelial progeny (Fig. 2A,B). Stem cells were not arrested, however, as they could incorporate BrdU in S-phase and the increased stem cell population showed continual cell division through G2/M-phases as marked by α-phosphorylated histone H3 on serine 10 (H3P) (Fig. 2C,D). TUNEL staining to measure cell death was relatively unchanged until the late stages of the time course when animals were closer to lysis and death, suggesting that progenitor cells are not dying, nor is the BAF complex required for cell survival (F4D9; Additional file 3A). Furthermore, at later timepoints (i.e., F4D6 and beyond), we began to see reductions of H3P+ cells and *piwi-1+* cells from their peaks, suggesting that these phenotypes are not maintained indefinitely. From these data, we concluded that differentiation was the primary defect following BAF complex disruption.

During the assays of the stem cell compartment in RNAi conditions, we observed an uncommon stem cell phenotype: detection of *piwi-1*+ cells anterior to the photoreceptors, which is normally almost devoid of stem cells (Fig. 2E). This could be due to patterning or migration defects, but could also be due to differentiating cells unable to turn off the *piwi-1* transcript and terminally differentiate. To test this, planarians were subjected to RNAi then FISH for *piwi-1* and co-stained with α-PIWI-1. Neoblasts express *piwi-1* and are PIWI-1 protein positive (*piwi-1+/*PIWI-1+), while newly made post-mitotic progenitors cease *piwi-1* expression, but PIWI-1 protein persists transiently (*piwi-1*-/PIWI-1+). Thus, proportions of *piwi-1+*/PIWI-1+ and *piwi-1-*/PIWI-1+ cells were quantified around the photoreceptors (Additional file 3B). In *control(RNAi)*, *piwi-1-*/PIWI-1+ cells were detected anterior and posterior to photoreceptors, while *piwi-1*+/PIWI-1+ were only posterior. In contrast, in *brg1(RNAi)* or *smarcc2(RNAi)*, both *piwi-1+*/PIWI-1+ and *piwi-1-*/PIWI-1+ were present anterior and posterior to photoreceptors, and there was a greater proportion of *piwi-1+/*PIWI-1+ cells (Additional file 3B), suggesting that immediate progeny are not turning off expression of *piwi-1* or that neoblasts are not transitioning into progenitors. From these data, we conclude that loss of the BAF complex does not cause stem cell loss or arrest, but causes inability of stem cells to differentiate. Thus, we next tested what cell lineages were disrupted, as well as how the genomic regulatory landscape changes in planarian stem cells when the BAF complex is knocked down.

### The BAF complex functions primarily to facilitate transcription

**Fig. 3:**
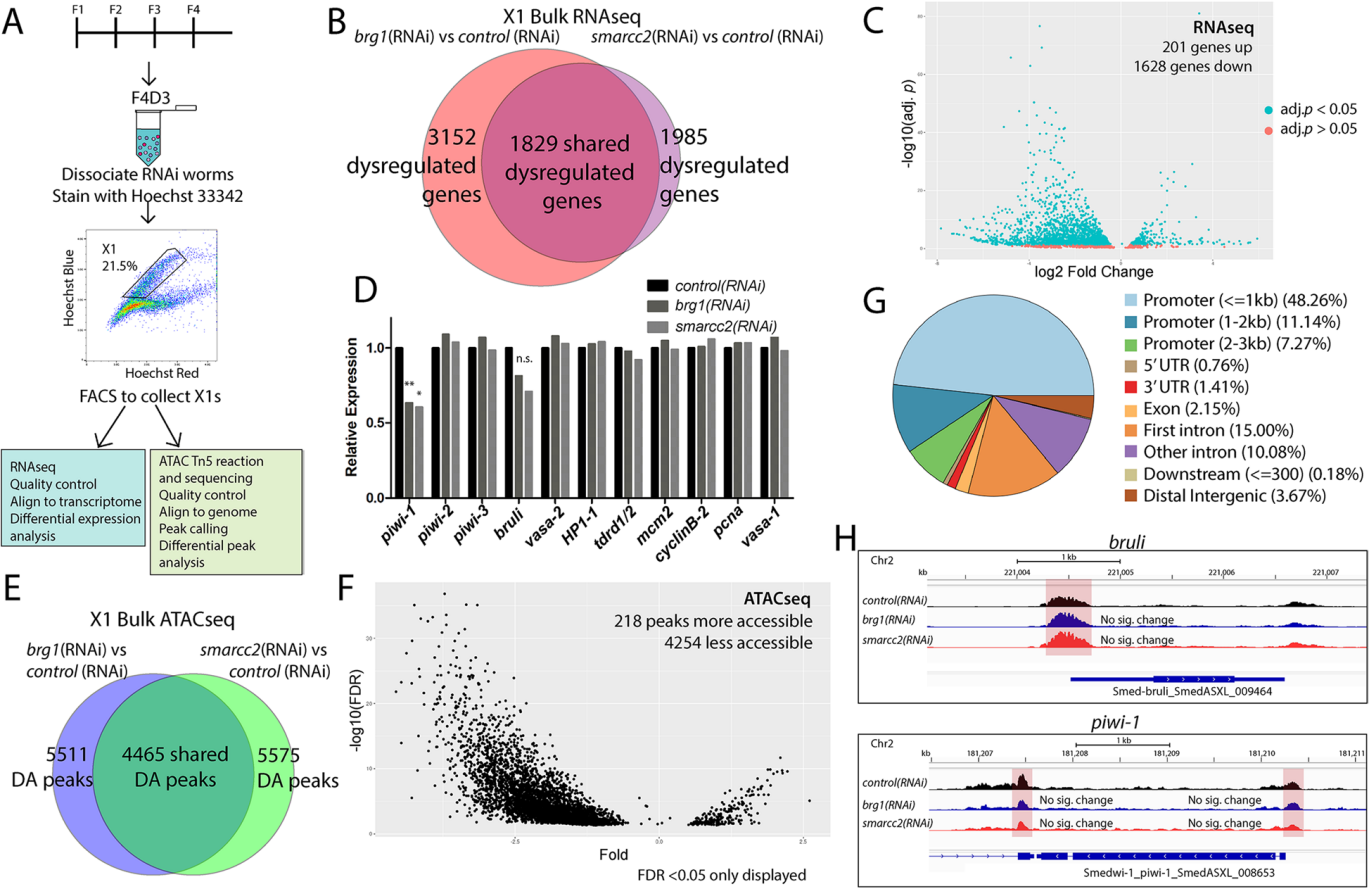
RNA and ATAC sequencing indicate the BAF complex is primarily a transcriptional activator. **A** Schematic of experimental process describing dissociation of worms and purification of X1s (neoblasts), processing for RNA or ATAC sequencing, and general downstream analyses. **B** Venn diagram demonstrating overlap of differentially expressed transcripts between *brg1(RNAi)* versus *control(RNAi)* and *smarcc2(RNAi)* versus *control(RNAi)* X1s (adj. *p* <0.05, no fold cut-off). **C** Volcano plot displaying commonly dysregulated transcripts between *brg1(RNAi)* versus *control(RNAi)* and *smarcc2(RNAi)* versus *control(RNAi)* X1s. Average log2 fold changes and adj. *p* values were used in plot. **D** Relative expression of neoblast-associated transcripts in *brg1*, *smarcc2*, or *control* RNAi in X1s. * adj. *p* < 0.05; **adj. *p* < 0.01. **E** Venn diagram demonstrating overlap of DA chromatin peaks between *brg1(RNAi)* versus *control(RNAi)* and *smarcc2(RNAi)* versus *control(RNAi)* X1s (FDR <0.05, no fold cut-off). **F** Volcano plot displaying commonly differentially accessible peaks shared between *brg1(RNAi)* versus *control(RNAi)* and *smarcc2(RNAi)* versus *control(RNAi)* X1s. Average fold changes and FDR values were used in plot. **G** Locations of differentially accessible peaks in X1s during *brg1* and *smarcc2* RNAi compared to *control* RNAi with respect to the nearest predicted gene. **H** ATACseq peaks identified in X1s during RNAi at *piwi-1* and *bruli* loci. The sequence coverage tracks show replicate-averaged, sequence depth-normalized (counts per million, CPM) read coverage for each RNAi condition

In yeast, flies, and mammals, the BAF/BAP complex is primarily a physical opener of chromatin, which facilitates activation of gene transcription [71–73]. We reasoned that because stem cells exhibit impaired differentiation but are not depleted themselves, we could specifically measure transcription and chromatin accessibility in stem cells at the bulk-population level to determine the key genes or pathways that become dysregulated to provide insights into how the BAF complex regulates differentiation. To this end, we used flow cytometry to purify the stem cell X1 gate and performed bulk RNAseq and bulk Assay of Transposase Accessible Chromatin (ATAC) sequencing in *control(RNAi)*, *brg1(RNAi)*, or *smarcc2(RNAi)* backgrounds (Fig. 3A; see “ Methods”). As noted, *brg1* and *smarcc2* were also highly expressed in the X2 population that contains G0/G1 stem cells and post-mitotic progenitors, so we performed RNAseq and differential gene expression analysis of X2s in all RNAi conditions (Additional file 4). However, because the X2 population contains multiple cell types and is not well-characterized, we focused on the X1 cell population for subsequent analyses and did not analyze the X2 RNAseq data further.

Using the bulk RNAseq data from stem cells, differential gene expression analysis was performed in *control(RNAi)* versus either *brg1(RNAi)* or *smarcc2(RNAi)* contexts, and 3152 and 1985 differentially expressed transcripts were identified, respectively (adj. *p*<0.05; Fig. 3B). Of the differentially-expressed transcripts, 1829 were commonly dysregulated between *control(RNAi)* versus *brg1(RNAi)* (57% of total differentially expressed transcripts) and *control(RNAi)* versus *smarcc2(RNAi)* (91% of total differentially expressed transcripts) contexts (Fig. 3B, Additional file 5), suggesting RNAi of either BAF complex core component is affecting expression of similar genes in neoblasts, as expected if these BAF subunits are in the same complex. Importantly, 89% of the common set of dysregulated genes were downregulated, consistent with the conserved function of the BAF complex as a transcriptional facilitator (Fig. 3C). In *brg1(RNAi)* or *smarcc2(RNAi)*, neoblasts increase in number, so neoblast genes should be robustly expressed. Expression of canonical neoblast and cell cycle genes was generally unchanged in *control*, *brg1*, or *smarcc2* RNAi conditions (Fig. 3D). Interestingly, we found that *piwi-1* expression was ~60% that of *control(RNAi)*. Although more cells were expressing *piwi-1* with disruption of the BAF complex (Fig. 2A), cells were expressing *piwi-1* at a lower level.

Differentially accessible (DA) chromatin regions were also compared across stem cells from *control(RNAi)* versus either *brg1(RNAi)* or *smarcc2(RNAi)* animals. There were 5511 and 5575 DA peaks in *brg1(RNAi)* or *smarcc2(RNAi)* contexts compared to *control(RNAi)*, respectively (FDR < 0.05). Of the ~5500 differentially accessible peaks, 4465 were shared between *brg1(RNAi)* (81% of DA peaks) and *smarcc2(RNAi)* (80% of total DA peaks) (Fig. 3E, Additional file 6). Of the 4456 shared DA peaks, 4244 (95%) were less accessible (Fig. 3F), consistent with the BAF complex being an opener of chromatin. DA peaks were assigned to their closest genes as putative cis-regulatory regions: 48% of DA peaks were within 1kb of the predicted transcription start site (TSS), and an additional 18% of DA peaks were within 1–3kb of the TSS. Further, 25% of DA peaks were intronic, and minor proportions were associated with 5′ or 3′ UTRs, exons, proximal downstream (3′ to the transcript), or distal intergenic (>3kb away) (Fig. 3G). Neoblast transcripts, including *piwi-1* and *bruli* [74], remained accessible in all conditions, supporting the phenotypic observations that stem cell maintenance itself is not changed with disruption of the BAF complex (Fig. 3H). Additionally, σ-neoblasts, a subtype of neoblasts capable of self-renewal with broad lineage capacity [51], were present at similar levels in *control*, *brg1*, or *smarcc2* RNAi contexts (Additional file 7A).

When trying to reconcile whether changes in transcript abundance correlated with changes in chromatin accessibility at their genomic loci in stem cells, we found that the 4465 shared DA peaks were associated with 3945 unique transcripts. Of these transcripts, 497 were determined to be dysregulated by RNAseq, totalling ~27% (497/1829) of all transcripts with significantly altered expression in stem cells in both *brg1* and *smarcc2* RNAi conditions (Additional file 7B). That is, a large fraction of transcripts that were downregulated in both *brg1* and *smarcc2* stem cells had putative regulatory regions that became less accessible at the chromatin level. DA peaks within 1kb to putative TSS’s of genes more frequently resulted in downregulation of the associated transcripts (20.6% of DA peaks) than DA peaks at other locations (12.2% of DA peaks) (Additional file 7C). Furthermore, downregulated genes with DA peaks within 1kb of the TSS were downregulated to a greater degree (average −2.57 log2 fold) compared to those in other locations (average −1.96 log2 fold) (Additional file 7D). Together, the RNA and ATAC sequencing data support a conserved role for the BAF complex in opening chromatin to facilitate transcription. We next examined how changes in chromatin accessibility to known fate-specific TFs could explain the cellular phenotypes that occur in *brg1* or *smarcc2* RNAi animals.

### The BAF complex is not required for gamma neoblasts nor gut lineages (endoderm)

**Fig. 4:**
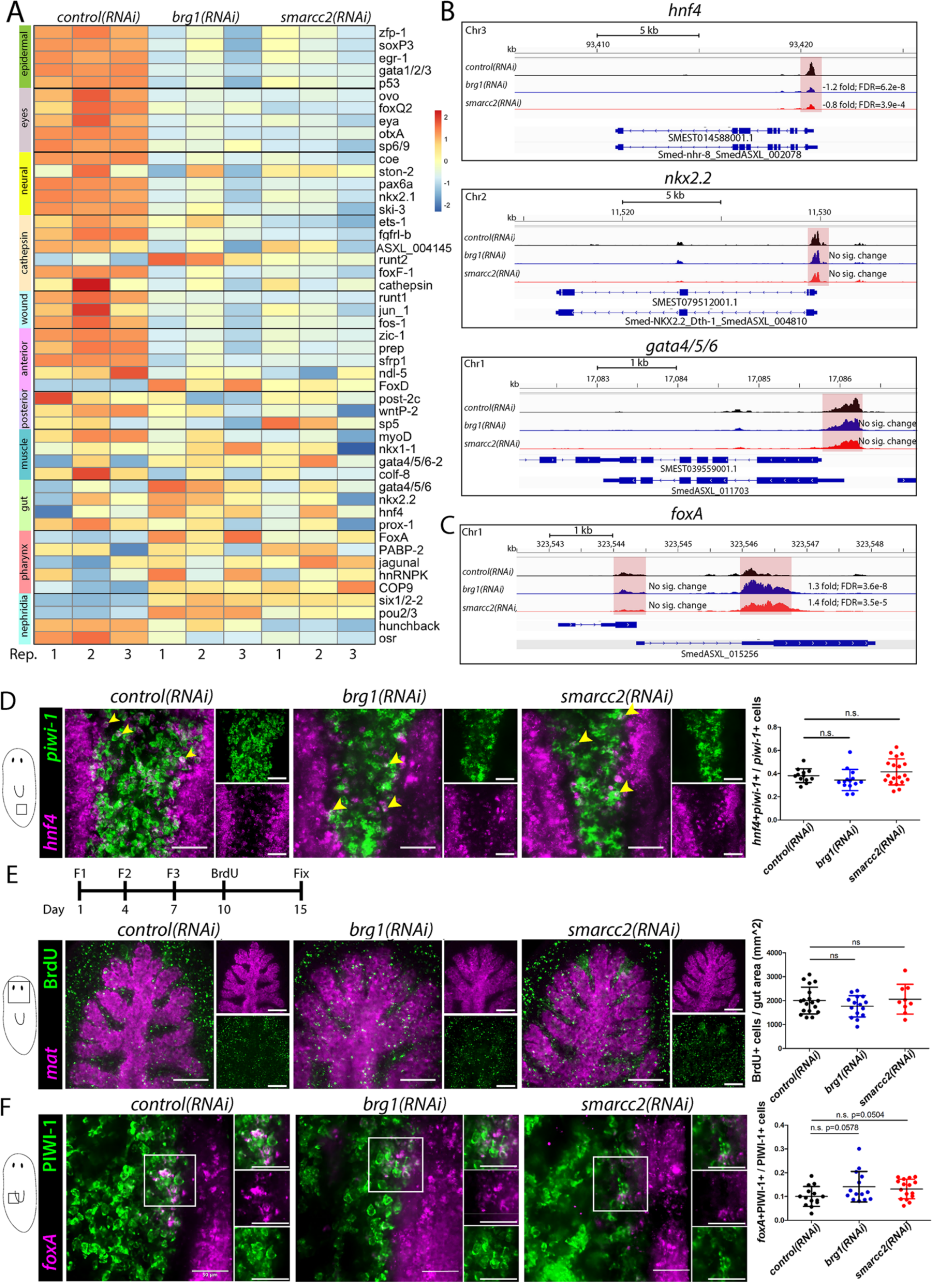
Gut and pharyngeal lineage TFs are accessible and expressed in *brg1* and *smarcc2* RNAi. **A** Heatmap of differentially expressed genes in X1s during RNAi. Displayed genes were selected based on their determined or postulated relevance to the lineage indicated on the label on the left. Replicates are displayed independently. **B** ATACseq peaks identified during RNAi in X1s at the *hnf4*, *nkx2.2*, *gata4/5/6* and *foxA* loci, associated with γ-neoblasts and pharynx progenitors. The sequence coverage tracks show replicate-averaged, sequence depth-normalized (CPM) read coverage for each RNAi condition. **C** dFISH of *piwi-1* and *hnf4* to mark γ-neoblasts in RNAi conditions at F4D3. Planarians were imaged at the boxed region indicated in the diagram to the left. *hnf4+piwi-1+* cells to total *piwi-1*+ cells are quantified in graph to the right. Scale bar is 50µm. **D** Immunostaining of BrdU with *mat* FISH for planarians subjected to experimental course indicated in the timeline above images. Planarians were imaged at the boxed region indicated in the diagram to the left. Quantifications of the number BrdU cells within the gut area (defined by *mat* expression) through five confocal slices are shown in graphs to the right. Scale bars, 100µm. **E** dFISH of *piwi-1* and *foxA* in RNAi conditions at F4D3. Planarians were imaged at the boxed region indicated in the diagram to the left. *foxA+piwi-1+* to total *piwi-1*+ cells are quantified in graph to the right. Scale bar is 50µm. Quantifications are mean $\pm$ 1 S.D Significance levels in plots: *p<0.05, **p<0.01, ***p<0.001, each “*n*” is a representative region from 1 planarian

To determine which genes, cell types, or lineages were affected by *brg1* and *smarcc2* RNAi, we first selected 4–6 critical genes known to be required for the generation of specific cell types of major tissues (Fig. 4A) [50–52, 57, 75–82]. Transcripts of genes required for generation of epidermal, neural, photoreceptor, and cathepsin lineages were generally and significantly downregulated as were genes expressed in response to injury and to specify the anterior pole. Genes required for posterior pole specification and muscle were not consistently dysregulated between conditions. Interestingly, genes required for protonephridial lineages were both upregulated and downregulated.

Transcripts expressed in γ-neoblasts, a neoblast subtype required for generation of the intestinal lineages, were not significantly affected by disruption of the BAF complex RNAi (Fig. 4A). Genomic loci associated with γ-neoblasts markers *hnf4*, *gata4/5/6*, and *nkx2.2* remained accessible in all experimental conditions (Fig. 4B) [51, 83]. Interestingly, a putative cis-regulatory region at the promoter of *hnf4*, which is expressed in both the cathepsin and intestinal lineages, is mildly but significantly less accessible. As mentioned, peaks associated with genes of the cathepsin lineage were less accessible and the transcripts were downregulated. Perhaps the BAF complex is required for chromatin accessibility at *hnf4* in stem cells fated towards cathepsin fates but not intestinal fates resulting in a mild change in accessibility in the bulk population level, although this needs to be explored further. Furthermore, the proportion of γ-neoblasts, marked by *hnf4+*/*piwi-1*+ cells, in the total neoblast population was unchanged (Fig. 4D). To ensure new gut tissue was being made, we performed a BrdU pulse followed by a 5-day chase in *control(RNAi)*, *brg1(RNAi*), or *smarcc2(RNAi)* planarians. There were no changes in the generation of new gut cells made as determined by number of BrdU+ cells/gut area (Fig. 4E), indicating that stem cell access to gut lineage differentiation does not require the BAF complex.

Genes associated with pharynx lineages were unchanged or significantly upregulated with disruption of the BAF complex (Fig. 4A). *foxA* is expressed in pharynx tissue and a subpopulation of neoblasts and is required for pharynx regeneration [50]. Upon *brg1* or *smarcc2* RNAi, peaks associated with *foxA* were significantly more accessible and transcript levels were significantly increased in X1s (Fig. 4A,C). *foxA+/*PIWI-1*+* cells, representing neoblasts and post-mitotic progeny with pharyngeal identity, were maintained at normal levels and in the correct spatial position despite disruption to the BAF complex (Fig. 4F)*.* Together, these data suggest that the planarian BAF complex was not required for generation of endoderm-derived lineages; however, genes associated with other lineages were downregulated. Thus, we next investigated how the BAF complex could regulate non-endodermal cell lineages.

### The BAF complex controls access to several mesodermal fates

**Fig. 5:**
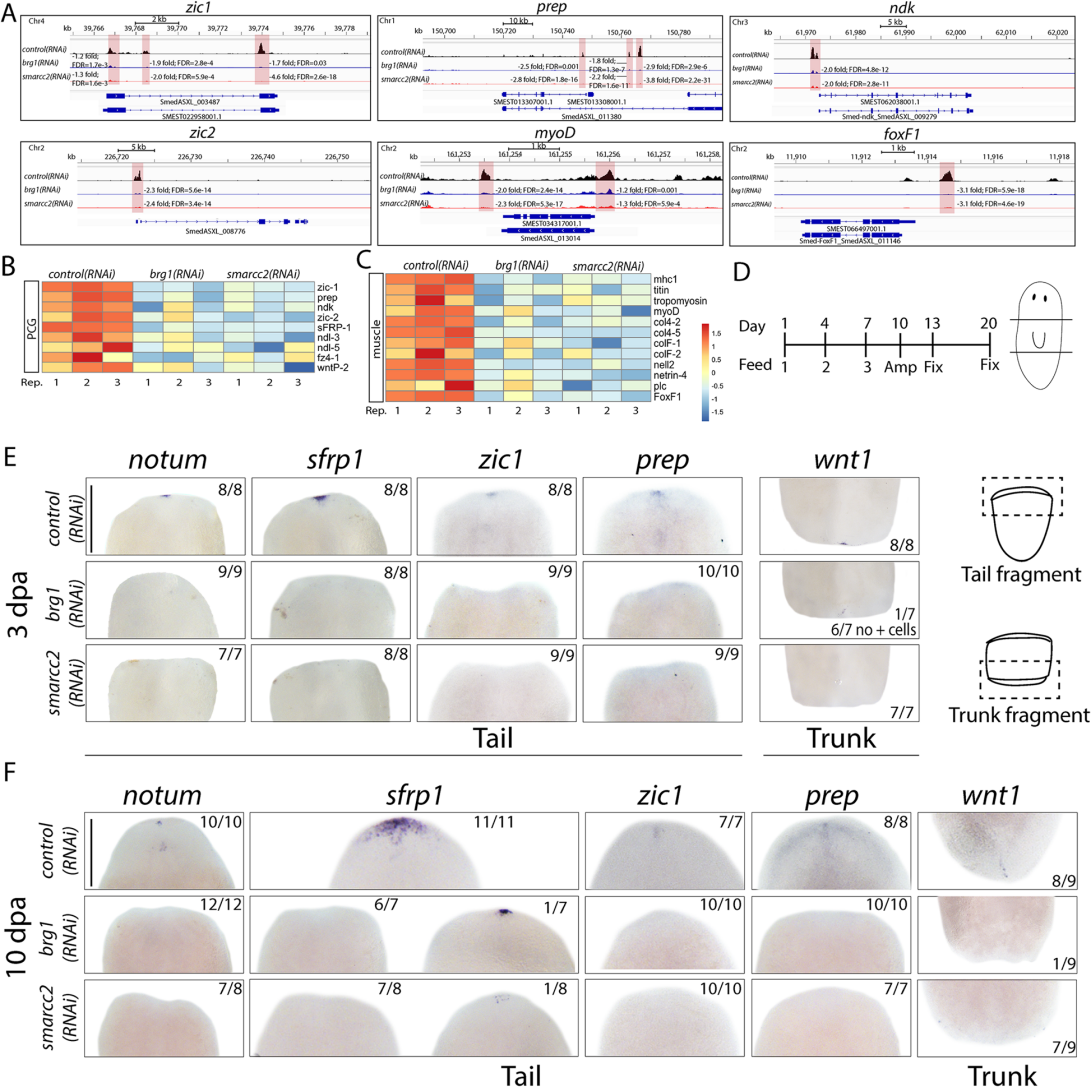
The BAF complex controls access to mesodermal fates and is required for pole regeneration. **A** ATACseq peaks during RNAi in X1s at PCGs (*zic1*, *prep*, *ndk*, and *zic2*) and muscle (*myoD* and *foxF1*) loci of mesodermal lineages. The sequence coverage tracks show replicate-averaged, sequence depth-normalized (CPM) read coverage for each RNAi condition. **B** Heatmap of differentially expressed PCGs from RNAi conditions in X1s. Replicates are displayed independently. **C** Heatmap of differentially expressed muscle-associated genes from RNAi conditions in X1s. Replicates are displayed independently. **D** Experimental timeline for regeneration experiments in (**E** and **F**). The planarian cartoon indicates the location of the amputation sites. **E** WISH of 3dpa tail and trunk fragments are shown, with the number of worms displaying that phenotype indicated, according to timeline in (**D**). Scale bars, 200µm. **F** Representative images of WISH of 10dpa tail and trunk fragments are shown, with the number of worms displaying that phenotype indicated, according to timeline in (**D**). Scale bars, 200µm. dpa is days post amputation

In the bulk RNAseq datasets, we noticed that genes associated with anterior pole and positional control muscle cells were downregulated in *brg1* and *smarcc2* RNAi (Fig. 4A). Specific muscle cells express positional control genes (PCGs) to maintain and regenerate patterning of new tissues and organs, and their disruption can cause the abolishment of regeneration similar to what we observed in *brg1* or *smarcc2* RNAi animals [49]. Our ATACseq datasets (derived from intact worms, but dissociated during processing for flow cytometry) indicated that PCGs, particularly anterior-pole genes such as *prep*, and *zic1* [78, 79], were less accessible in neoblasts upon disruption of the BAF complex (Fig. 5A). Genes involved in body wall and non-body wall muscle cell generation, *myoD* and *foxF-1* [75, 84], were less accessible with disruption of the BAF complex (Fig. 5A). Furthermore, stem cell RNAseq data demonstrated that PCGs and muscle-related genes, including *prep*, *zic-1*, and *foxF-1*, were downregulated in both *brg1(RNAi)* and *smarcc2(RNAi)* neoblasts (Fig. 5B,C) [82]. To determine whether downregulation of genes critical to the anterior pole in intact planarian stem cells actually resulted in less anterior pole cells during regeneration, we assayed anterior pole markers *notum*, *sfrp1*, *zic1*, *prep*, and the posterior marker *wnt1* at 3 and 10 days of regeneration in each RNAi condition [79, 85, 86]. Although *notum* was not downregulated in our *brg1* or *smarcc2* neoblast datasets, its expression would distinguish whether the pole can regenerate or not. We observed that *prep*, *zic1*, and *sfrp1* were downregulated in *brg1(RNAi)* or *smarcc2(RNAi)* regenerating tail fragments and the *notum+* pole was absent (Fig. 5D–F)*.* From these data, we concluded that the BAF complex is required to access specific muscle fates.

Single-cell RNAseq cell atlases in planarians have identified another major cell lineage, the “cathepsin lineage,” marked by the transcript *cathepsin-L1*. While this lineage is largely unexplored, it contains glial, pigment, and immune-like cell types, and from this, we believe it may have a mesodermal identity [57, 58, 87]. Key regulators of this lineage have been shown to be the *ets-1* and *foxF-1* TFs. In our data, *ets-1* and *foxF-1* were significantly less accessible, and the transcripts were also downregulated in both *brg1* and *smarcc2* RNAi (Fig. 5A, Additional file 8A-B). Consistent with this, neoblasts and immediate post-mitotic progeny expressing the cathepsin lineage marker *ets-1* (*ets-1+*/PIWI-1+ cells) were reduced in *brg1(RNAi)* or *smarcc2(RNAi)* conditions compared to *control(RNAi)* (Additional file 8C). Together, these data suggest that the planarian BAF complex regulates stem cell access to specific cell lineages for multiple mesodermal cell types.

### The BAF complex is required for zeta-neoblasts and controls access to epidermal fates (ectoderm)

**Fig. 6:**
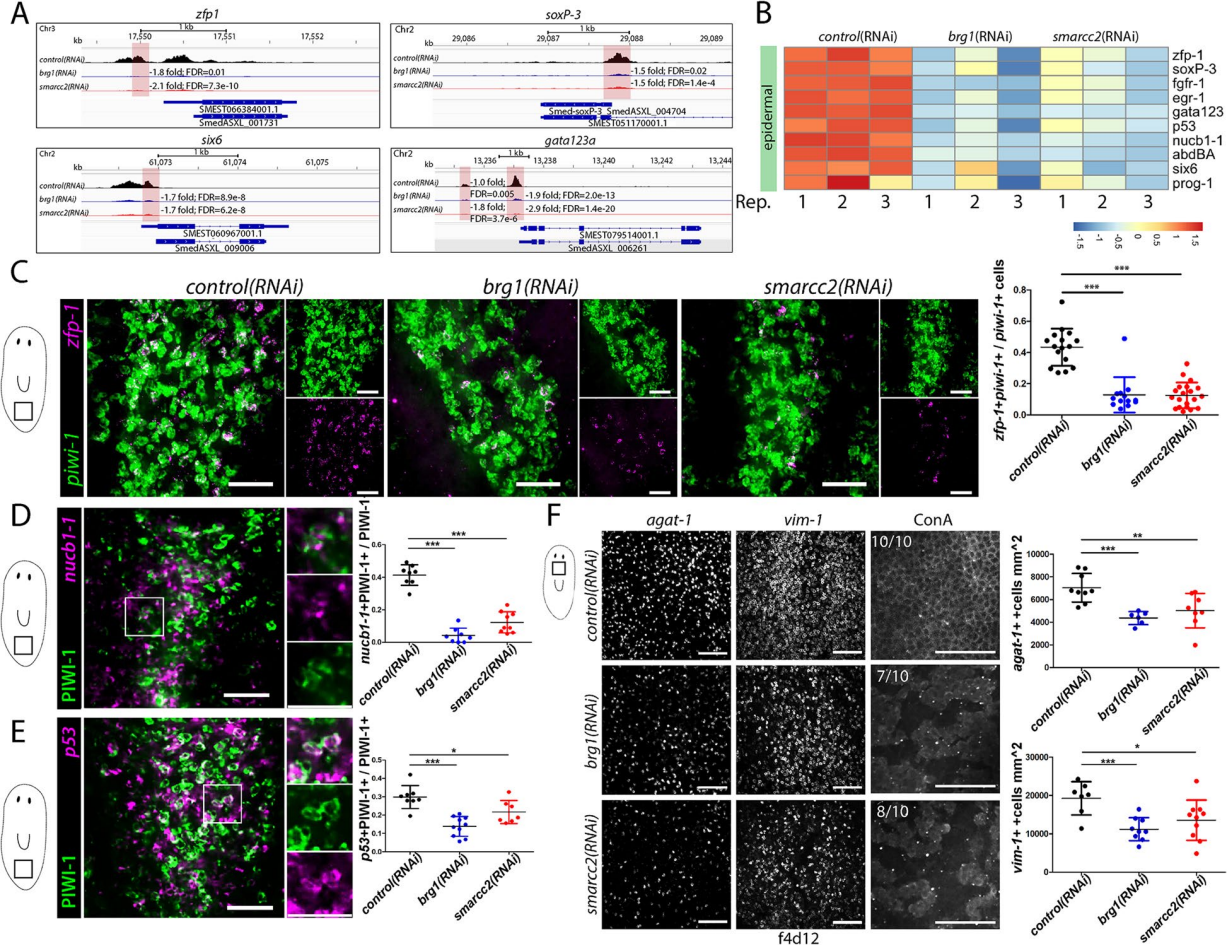
The BAF complex is required for stem cell access to the epithelial lineage. **A** ATACseq peaks during RNAi in X1s at loci of key regulators of the epidermal lineage. The sequence coverage tracks show replicate-averaged, sequence depth-normalized (CPM) read coverage for each RNAi condition. **B** Heatmap of differentially expressed epithelial associated genes during RNAi conditions in X1s. Replicates are displayed independently. **C** dFISH of *piwi-1* and *zfp-1* during RNAi at F4D3. Planarians were imaged at the boxed region indicated in the diagram to the left of the images. *zfp-1+piwi-1+* cells to total *piwi-1*+ cells are quantified in graph to the right. Scale bars, 50µm. **D** Immunostaining of α-PIWI-1 with FISH for *nucb1-1* during RNAi. Representative image demonstrating colocalization in *control(RNAi)* conditions is shown. Planarians were imaged at the boxed region indicated in the diagram to the left of the images and quantified within the *nucb1-1* dorsal expression domain. *nucb1-1+*PIWI-1*+* cells to total PIWI -1+ cells in RNAi conditions at F4D3 are quantified in graph to the right Scale bars, 50µm. **E** Immunostaining of α-PIWI-1 with FISH of *p53* during RNAi. Representative image demonstrating colocalization in *control(RNAi)* conditions is shown. Planarians were imaged at the boxed region indicated in the diagram to the left of the images and quantified within the *p53* dorsal expression domain. *p53+*PIWI-1*+* cells to total PIWI-1+ cells in RNAi conditions at F4D3 are quantified in graph to the right. Scale bars, 50µm. **F** dFISH of late epidermal progenitor marker *agat-1* and mature epidermal marker *vim-1*, as well as Concanavalin-A (ConA), which binds to cell membranes in RNAi conditions, at F4D12. Planarians were imaged in the boxed region indicated in the diagram to the left of the images and dorsal staining was quantified. Representative images are shown (*n*>8). Scale bars, 50µm. Quantifications are mean $\pm$ 1 S.D Significance levels in plots: *p<0.05, **p<0.01, ***p<0.001, each “*n*” is a representative region from 1 planarian

Consistent with the loss of *prog-2*+ cells observed during RNAi of *brg1* or *smarcc2* (Fig. 2B), genes required for specification and differentiation along the epidermal lineage, including *zfp-1*, *soxP-3*, *six6*, and *gata1/2/3a* [42, 51, 84], were less accessible upon disruption of the BAF complex (Fig. 6A). For instance, *zfp-1* expression in neoblasts specifies ζ-neoblasts and is required for generation of the epidermal lineage [51]. We find that disruption of the BAF complex reduced accessibility of *zfp-1* putative cis-regulatory elements in neoblasts (Fig. 6A). Furthermore, TFs required for epidermal lineage generation, including *zfp-1*, *six6*, *gata123a*, *p53*, and *soxP-3*, as well as other epidermal lineage-associated genes such as *nucb1-1* and *prog-1* were downregulated in neoblasts in BAF RNAi (Fig. 6B) [38, 84, 88].

We performed dFISH for *zfp-1* and *piwi-1* to assess the ζ-neoblast population upon *brg1(RNAi)* or *smarcc2(RNAi)*, and as expected, ζ-neoblasts were significantly reduced (Fig. 6C). Co-expression of *nucb1-1* or *p53* with α-PIWI-1 was also reduced in *brg1(RNAi)* or *smarcc2(RNAi)* conditions compared to *control(RNAi)*, supporting the impaired generation of new epidermal-specified cells (Fig. 6D,E). *agat-1+* cells representing late epidermal progenitor cells and *vim-1+* cells representing mature epidermal cells were reduced as the *brg1(RNAi)* or *smarcc2(RNAi)* phenotypes progressed (Fig. 6F). Finally, when RNAi animals were stained with fluorescently-labeled Concanavalin A (ConA), which binds to cell membranes, there was a clear disruption to the dorsal epidermal layer in BAF RNAi conditions (Fig. 6F). Together, the expression and accessibility data, as well as the epidermal cell-lineage analyses, supported the hypothesis that the BAF complex is required for stem cells to access the epidermal lineage.

### The BAF complex is required for *tgs-1*+ neoblasts and controls access to neural fates (ectoderm)

**Fig. 7:**
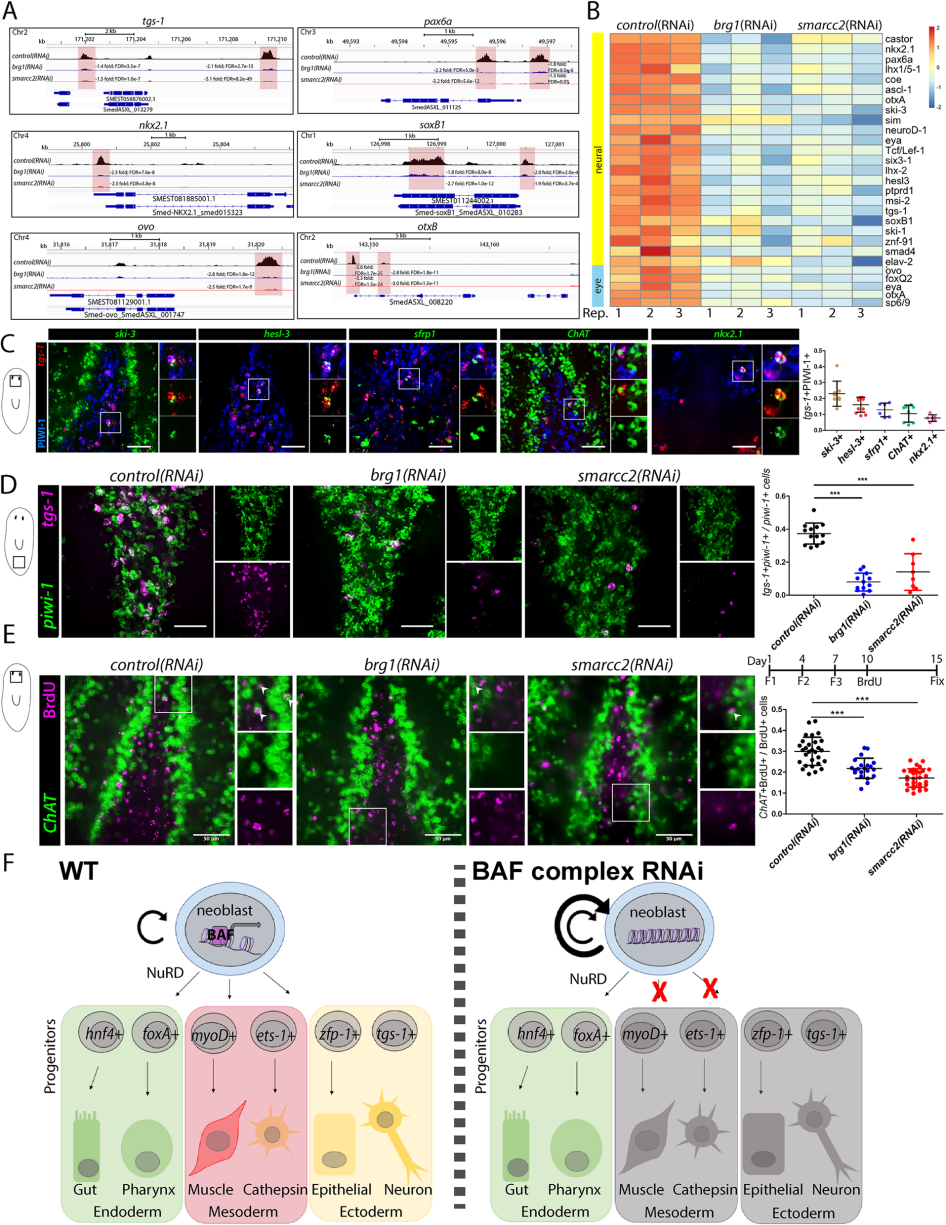
The BAF complex regulates accessibility of *tgs-1* and loci associated with neural fates. **A** ATACseq peaks identified in RNAi conditions of X1s at loci associated with known regulators of neural fates. The sequence coverage tracks show replicate-averaged, sequence depth-normalized (CPM) read coverage for each RNAi condition. **B** Heatmap of differentially expressed neural-associated genes from RNAi conditions X1s. Replicates are displayed independently. **C** dFISH of *tgs-1* and the gene indicated above, and immunostaining of α-PIWI-1 in wildtype planarians. Planarians were imaged at the boxed region indicated in the diagram to the left of the images. Cells expressing the *gene* indicated (green), *tgs-1* (red), and PIWI-1 (blue), to total *tgs-1+*PIWI-1+ cells are quantified in the graph to the right. Scale bar is 50µm. **D** dFISH of *piwi-1* and *tgs-1* in RNAi conditions at F4D3. Planarians were imaged at the boxed region indicated in the diagram to the left of the images. *tgs-1+piwi-1+* to total *piwi-1+* cells are quantified in the graph to the right. Scale bar is 50µm. **E** Immunostaining of BrdU with FISH for *ChAT* in planarians subjected to the RNAi for the indicated gene. Planarians were imaged at the boxed region indicated in the diagram to the left. *ChAT+*BrdU+ to total BrdU+ cells are quantified in graph to the right. Scale bars, 50µm. **F** Model of how the BAF complex functions in planarian stem cell biology to allow access to specific mesodermal and ectodermal fates whereas the route to endodermal fates requires the NuRD complex. Quantifications are mean $\pm$ 1 S.D Significance levels in plots: *p<0.05, **p<0.01, ***p<0.001, each “*n*” is a representative region from 1 planarian

*tgs-1*+ neoblasts have been reported to possess enriched pluripotency [53]. However, scRNAseq of X1 neoblasts in G2/M phases revealed that *tgs-1+* neoblasts also co-express neural fate-specific TFs, as well as some markers of the anterior pole, such as *sfrp-1* [59]. In *brg1* or *smarcc2* RNAi neoblasts, we found *tgs-1* was significantly downregulated and its putative regulatory regions were less accessible (Fig. 7A,B), thus, we further investigated this neoblast subpopulation and other key neural-associated genes. Strikingly, 35/50 of the top genes enriched in *tgs-1*+ neoblasts were downregulated and 20 had less accessible genomic loci by ATACseq in *brg1(RNAi)* and *smarcc2(RNAi)* neoblasts (Additional file 9). Some of the genes with reduced chromatin accessibility or expression in *brg1* or *smarcc2* RNAi stem cells, such as *soxB1, neuroD-1,* and *hesl3*, are TFs that control neural cell fates. We observed that *tgs-1+/*PIWI-1+ cells colocalized with *ski-3*, *hesl-3*, and *sfrp-1* in situ (Fig. 7C). *hesl3* and *ski-3* are implicated in neural lineage generation [52, 89] and *sfrp-1* is expressed in muscle cells of the anterior pole [86]. Further, *tgs-1+/*PIWI-1+ cells colocalized with neural associated gene *ChAT* and the neural TF *nkx2.1* (Fig. 7C) [90]. Upon *brg1(RNAi)* or *smarcc2(RNAi)*, *tgs-1+/piwi-1+* neoblasts were significantly reduced compared to *control(RNAi)* (Fig. 7D)*.* Together, these data suggested that the BAF complex was required to generate the *tgs-1+* neoblast state which likely contributes to neural lineages and anterior pole cells.

In addition to the known neural factors that are co-expressed in *tgs-1*+ neoblasts, we observed that other TFs associated with neural lineages were transcriptionally downregulated and had reduced chromatin accessibility at putative genomic regulatory elements in stem cells in *brg1* or *smarcc2* RNAi. The genes included key regulators of multiple neural tissues such as the brain (*tcf-1*, *coe*, *pax6a*, *otxB*), sensory neurons (*soxB1*), and eyes (*ovo*, *eya*, *otxA*, *sp6/9*). Many of the affected genes have been shown to be required for generation of specific neural subtypes in planarians, where their RNAi results in defective neurogenesis and/or regeneration [69, 90–94], suggesting that these processes may be affected with disruption of the BAF complex. To understand how the BAF complex regulates neural lineage production in vivo, we assayed *ChAT*+/PIWI-1+ cells in *brg1* or *smarcc2* RNAi conditions. *ChAT* is broadly expressed in mature neurons and a subpopulation of *piwi-1*+ cells [42]. Following *brg1(RNAi)* or *smarcc2(RNAi)*, *ChAT+*/PIWI-1+ cells were significantly reduced (Additional file 10A). Furthermore, when BrdU was administered to RNAi animals and allowed 5 days to chase into *ChAT*+ cells to measure rates of neuronal production, we found reduced neurogenesis with disruption of the BAF complex (Fig. 7E).

We next assayed the proportion of neoblast and immediate progenitors expressing neural TFs that were downregulated by RNAseq and less accessible by ATACseq in both *brg1(RNAi)* and *smarcc2(RNAi)* stem cells: *pax6a*, *tcf-1*, *coe*, *nkx2.1*, *otxb*, and *ski-3*. Consistent with the sequencing data, PIWI-1+ cells expressing *pax6a*, *tcf-1*, *coe*, *nkx2.1*, *otxB*, or *ski-3* were less frequent in *brg1(RNAi)* or *smarcc2(RNAi)* compared to *control(RNAi)* worms (Additional file 10B). Finally, we assayed *ovo*+ eye progenitors and found that they were ablated following RNAi of the BAF complex (Additional file 10C). In total, the genomic and phenotypic data with disruption of the BAF complex demonstrated key regulatory roles for the BAF complex to license planarian neoblasts to access a wide array of neural cell fates.

### Motif analysis of BAF-controlled DNA elements identifies TFs that may target the BAF complex

In general, SWI/SNF chromatin remodeling complexes do not bind DNA on their own, but are targeted to DNA by TFs, which have their own DNA binding site preferences. We reasoned that it may be possible to predict what broad family of TFs may target the BAF complex to its target loci in planarian stem cells through de novo binding site discovery using the HOMER pipeline, which finds statistically more frequent binding sites in DNA peaks versus the background rate across the whole genome [95]. We used only DA chromatin peaks that were significantly closed in both *smarcc2* and *brg1* RNAi stem cells within 1kb of a predicted TSS. The analysis found 21 enriched binding sites from known TF families, and interestingly included CUX1 and PAX6—both of which are expressed in *tgs-1*+ and nu-neoblasts (Additional file 11). In the future, it will be interesting to test whether any of the identified transcription factors families have phenotypes similar to BAF RNAi when knocked down and whether they bind the predicted DNA elements in planarian stem cells.

## Discussion

Here, we utilize ATACseq and RNAseq of planarian stem cells in a BAF complex-deficient background to understand how the BAF complex licenses neoblasts access to specific routes of differentiation. It was known that RNAi to essential BAF complex subunits resulted in disruption of epithelial differentiation, however, we were surprised to find that when we assayed transcriptomes and accessible DNA in stem cells, dysregulated genes fell into specific categories. We found that endodermal lineages did not depend on BAF complex function, yet some muscle, parenchymal cathepsin, and neural lineages did. Surprisingly, chromatin at key anterior pole genes required the BAF complex to remain open in uninjured planarians, and following amputation, anterior poles could not regenerate in *brg1* or *smarcc2* RNAi. This result suggests that, as a population, planarian stem cells at steady state have access to pole factors, even in a non-regenerative context. Finally, *tgs-1*+ neoblasts and neural factors became inaccessible in *brg1* and *smarcc2* RNAi. We demonstrate that the *tgs-1*+ cell state is lost in BAF RNAi and neural differentiation is also decreased. Considering *tgs-1*+ neoblasts co-express known neural genes, we suggest that BAF complex function is required for neural differentiation through a *tgs-1*+ stem cell state. In total, we put forth a model that BAF activity is required for planarian stem cells to access mesodermal and ectodermal cell fates (Fig. 7F). Our study furthers the understanding of how potency in planarian ASCs is regulated and can provide insights into BAF complex function in other systems.

A striking component of the *brg1* and *smarcc2* RNAi phenotypes is increased *piwi-1+* stem cells. It is reasonable that, with failed progression down numerous lineages plus an imbalance between differentiation and self-renewal, neoblasts accumulate. These neoblasts still proliferate and incorporate BrdU, suggesting they progress through the cell cycle; however, key genes required for differentiation through the mesodermal and ectodermal lineages are not activated and neoblasts ultimately self-renew. Interestingly, endoderm lineages are minimally affected with disruption of the BAF complex, suggesting differentiation down these lineages are regulated differently. It was recently discovered that RNAi of a catalytic subunit of the NuRD chromatin remodeling complex, *CHD4*, results in reduced expression of TFs required for intestine cell differentiation, as well as other endoderm-associated transcripts [96]. Together, this suggests a scenario where stem cells require activity of the BAF and NuRD complexes to enable access to key TFs that regulate differentiation into major cell lineages (Fig. 7F).

### Do *tgs-1*+ neoblasts give rise to neurons?

Although the functional heterogeneity of neoblasts is hotly contested, there are molecularly distinct subclasses or cell states of neoblasts. There is also a functional correlation that when a subclass of neoblasts is lost, the lineage is lost. In *brg1* or *smarcc2* RNAi, we find that both ζ-neoblasts and *tgs-1+* neoblasts were the most affected. *tgs-1*+ stem cells are an elusive subclass, with functional evidence suggesting they are enriched in pluripotency [53] and more recently described to be enriched for neural TFs and some anterior pole genes [59], which we demonstrate here in situ. In mammals, the neural progenitor BAF complex has important roles in neural stem cells and neural development in general [25, 97, 98], and from our data, we suggest this to be conserved in planarians. In *brg1* or *smarcc2* RNAi, we observed a corresponding downregulation of genes enriched in *tgs-1*+ neoblasts [59] as well as a reduction of neoblasts and progenitor cells expressing known neural TFs, suggesting that this cell lineage is reduced or missing (Fig. 7). Although still not a direct implication in neurogenesis, we find it likely that *tgs-1+* neoblasts produce at least some neural fates. Further, the relationship between the previously identified υ-neoblasts [52] and *tgs-*1+ neoblasts [59] is not clear—these neoblast subtypes commonly express some neural-related TFs and could represent overlapping subtypes, different cell states of a neoblast subtype, or distinct neural-fated neoblast subtypes that give rise to separate neural lineages. Discerning between these options requires identification of reliable subtype markers and key factors that regulate neurogenesis. Together, *tgs-1*+ neoblasts may represent two subclasses that contribute to neural lineages and another that is pluripotent, where BAF is a key regulator of both [53, 59, 99].

### How is BAF targeted to specific enhancers in planarian stem cells?

Members of the SWI/SNF group of chromatin remodelers are in large complexes that do not typically bind DNA on their own, so they must be targeted by TFs that do. In yeast, GAL4, a TF containing a DNA-binding domain and a transactivation domain, was found to recruit SWI/SNF to specific sites for chromatin remodeling [100, 101]. Since that initial discovery, many TFs with transactivating domains were found to interact with the BAF complex and recruit the complex to distinct genomic sites. For instance, the GATA factors can recruit the BAF complex to loci during heart development [28, 102], MYOD can recruit BAF to target genes in myoblasts [103], and SOX2 and OCT4 can recruit BAF to target loci in embryonic stem cells [17, 104]. Because TFs, their binding motifs, and the BAF complex are conserved, we predict that planarian TFs direct BAF to specific genomic loci to activate transcription in neoblasts. To this end, we used the software HOMER [95] to examine DA chromatin peaks controlled by both *brg1* and *smarcc2*, for all peaks within 1kb of a TSS in our data. HOMER uses known consensus binding motifs to look for any statistically over-represented sites in BAF-controlled peaks compared to the rest of the genome. This de novo TF binding analysis identified several tantalizing TFs such as PAX6 and CUX1—TFs expressed in nu-neoblasts and *tgs-1*+ neoblasts (Additional file 11). These will be top candidates to test in the future. It is still unclear what TFs act to target BAF to initiate stem cell lineage specification, but with ATACseq data, it is now possible to narrow down and test potential factors and recent work as begun to put together gene regulatory networks for planarian stem cells and identify potential enhancers [105]. In the future, it will be important to examine chromatin accessibility at the single-cell level and in different cell populations in order to understand the gene regulatory networks that allow differentiation across all cell lineages and during regeneration.

## Conclusions

How adult stem cells balance self-renewal and potency to differentiate into multiple cell lineages remains an important question in stem cell research. Somatic stem cells in adult organisms are particularly elusive due to challenges in studying them in common laboratory model systems. Here, using the planarian *Schmidtea mediterranea*, we provide novel insights into ASC regulation by the BAF chromatin remodeling complex. We demonstrate that the BAF complex acts to facilitate transcription by granting access to target genes in planarian ASCs. Furthermore, we observe that the BAF complex acts at key loci to regulate ectodermal and mesodermal cell fates, which in turn results in impaired ASC output of these lineages resulting in tissue dysregulation and planarian death. Together, we demonstrate the importance of chromatin accessibility in ASC regulation and suggest that the BAF complex is an important contributor to cell fate decisions. As we learn more about the specific loci, TFs, and chromatin remodelers that function in planarian stem cells, we are closer to understanding how pluripotency is maintained and how access to specific cell lineages is achieved, which has implications across stem cell biology in any system.

## Methods

### Planarians

Asexual clonal populations of *Schmidtea mediterranea* (strain CIW4) were maintained under standard laboratory conditions, as previously described [47, 106]. Planarians were maintained at 18°C in milliQ-polished water supplemented with 0.21g/L Instant Ocean, 0.1 mM KCl, 0.1 mM MgSO4, 0.12 mM NaHCO3, and fed calf liver paste approximately once per week.

### Planarian cloning and RNA interference

Planarian transcripts were cloned using forward and reverse primers into a double-stranded RNA (dsRNA) expression vector pT4P as previously described [48]. dsRNA-expressing HT115 cultures were prepared using pT4P clones, mixed with homogenized calf liver, and fed to animals as previously described [47]. Briefly, bacteria were grown to an O.D. 600 of 0.8–1.0 and induced with 1 mM isopropyl β-D-1-thiogalactopyranoside (IPTG) for 2 h. Bacteria were pelleted and mixed with liver paste at a ratio of 500μl of liver per 100ml of original culture volume. Animals were fed every 3 days for RNAi. In all cases, an RNAi vector with the GFP coding sequence was used as a negative control. All animals used for experiments were approximately 3–5mm in length and size-matched between experimental and control worms.

### Planarian gene expression analysis

Expression of BAF subunit homologs in FACS-isolated cell populations were determined from previously published data bulk RNA sequencing [43]. t-SNE plots representing expression of BAF subunit homologs in single cells were generated using freely available Digiworm (https://digiworm.wi.mit.edu/) [57]. Proportion of all cells or *piwi-1*+ cells expressing *brg1* or *smarcc2* was determined using the R package Seurat [107] and the raw single-cell sequencing data [57], where the number of cells expressing *brg1* or *smarcc2* was compared to the number of all cells or the number of cells expressing *piwi-1* (*gene*expression>0).

### Planarian WISH, FISH, TUNEL, BrdU and immunostaining

Riboprobes were made from PCR templates from the same vector used for RNAi above (pT4P) [108]. WISH and FISH were performed as previously described [108–110]. Briefly, 5% N-acetylcysteine (NAC) in phosphate-buffered saline (PBS) was used to kill the worms and remove mucus, followed by fixation in 4% formaldehyde in PBST (0.3% Triton-X) for 20 min. Worms were then rinsed with PBST, further permeabilized with Reduction solution (50mM DTT, 1% NP-40, 0.5% SDS, in PBS) for 3–5min at RT, and dehydrated with ethanol. Worms were bleached with 6% hydrogen peroxide (in ethanol) overnight and rehydrated with PBST. For ISH, worms were pre-hybridized for 2 h, and then hybridized with probe overnight at 56°C. Blocking solution (10% horse serum in MABT) was used for blocking and antibody incubation. Colorimetric stains were developed using 4-nitro blue tetrazolium chloride (NBT, Roche 11383213001) with 5-bromo-4-chloro-3-indolyl-phosphate (BCIP, Roche 11383221001). FISH stains were developed with either tyramide amplification, or Fast Blue B Salt (Sigma D9805) with naphthol AS-MX phosphate (Sigma 855). For immunostaining, rabbit anti-H3ser10p was used at 1:1000 (EMD Millipore 05-817R-I), or mouse anti-PIWI-1 (kind gift of Dr. Jochen Rink) was used at 1:1000 [49]. Secondary horseradish peroxidase (HRP)-conjugated anti-rabbit or anti-mouse were used at 1:200, with subsequent tyramide amplification. For H3P immunostaining without in situ and Concanavalin A, animals were killed in N-acetylcysteine and treated for 2 h with Carnoy’s fixative (6:3:1 ethanol:chloroform:acetic acid). Worms were then bleached, blocked, and immunostained as above. FITC-conjugated Concanavalin A (Vector Laboratories, VECTFL1001) was used at a concentration of 5 μg/mL, incubated overnight in blocking buffer.

Planarians for TUNEL were processed using 5% NAC and 4% formaldehyde as described above for ISH. TUNEL was performed as previously described [111, 112]. Briefly, worms were labeled with DIG-11-dUTP using terminal deoxynucleotidyl transferase for 4 h at 37°C (Thermo, EP0162), and then detected with anti-DIG-POD (1:1000) and tyramide amplification.

BrdU (Sigma-Aldrich B5002) labeling was performed as previously described [47]. Briefly, worms were adapted to a high-salt medium (5 g/L Instant Ocean) for 1–3 days prior to BrdU delivery, and thereafter maintained in high salt for the duration of the chase period. BrdU dissolved in 50% DMSO was fed at a concentration of 10mg/mL in liver paste at indicated time points. Fixation and FISH were performed as described above. After all steps of FISH were completed, worms were incubated in acid for 45 min (2N HCl, 0.5% Triton-X), then neutralized with 0.1M sodium borate. Blocking solution (10% BSA, 5mM thymidine, in PBST) was used for blocking and antibody incubation. BrdU was detected with mouse anti-BrdU at 1:300, followed by anti-mouse HRP (1:500) and tyramide amplification. Only worms with robust staining throughout the body were quantified.

### Microscopy and image acquisition, processing, and statistical analyses

Live worms, colorimetric WISH stains, H3P, and TUNEL were imaged on a Leica M165 fluorescent dissecting microscope with a Leica DFC7000T digital camera. FISH, anti-PIWI-1 immunostaining, and ConA staining were photographed with a Quorum Spinning Disk Confocal (Olympus IX81 microscope and Hamamatsu C9100-13 EM-CCD camera). Raw images were captured at 10X or 20X at a consistent region of the animal using Volocity software. Unless otherwise noted, animals were imaged dorsally, with their anterior end pointing up. The freely available ImageJ software (https://imagej.nih.gov/ij/) was used for cell quantifications, which, in most cases, cells was performed manually using the cell counter plugin. H3P+ cell quantifications were made using ImageJ, but were automated by converting the image to 8-bit binary, subtracting the background, then using the Analyze Particles function (size =2-infinity, circularity=0-1). Images were post-processed in Adobe Photoshop and figures assembled in Adobe Illustrator. Linear adjustments (brightness and contrast) were made for images of animals labeled by WISH, FISH, and immunostaining in order to best represent actual results. These adjustments were identical within a given experiment where comparisons were drawn between conditions.

All graphs were generated using GraphPad Prism software or Microsoft Excel. Two-tailed Student’s *t* test was performed between two groups (i.e., *control(RNAi)* vs *brg1(RNAi)* and *control(RNAi)* vs *smarcc2(RNAi)*). Information on biological replicates are included in figure legends or indicated in the plotted data. For example, *N*=10 indicates 10 individual planarians were assayed with either the whole worm analyzed or a representative, consistent region of the worm (imaged region indicated by cartoons beside figures).

### RNA sequencing and differential expression analysis of planarian cells

The planarian stem cell (X1) fraction was isolated after F4D3 *control(RNAi)*, *brg1(RNAi)*, or *smarcc2(RNAi)* by fluorescence activated cell sorting (FACS) as previously described [52, 88]. Approximately 500,000 X1 cells from >100 animals were isolated at on a Becton–Dickinson FACSAria. Total RNA was purified using Trizol (Invitrogen, 15596026), and poly-A-selected cDNA libraries were prepared using the TruSeq RNA library prep kits (Illumina, RS-122-2001). For RNA-seq, 100 base pair single-end reads were sequenced to a depth of >30 million and multiplexed on an Illumina NovaSeq for each condition. RNAseq was performed in triplicate (Additional files 4 and 5).

Adapter sequences were removed using trimmomatic [113]. Salmon was used to align reads to the Smed_ASXL transcriptome [47] (NCBI Bio Project PRJNA215411) and quantify transcript expression [114] and the R package DESeq2 (v 1.30.1) [115] was used to analyze differences in gene expression between *control(RNAi)* and *brg1(RNAi)* as well as *control(RNAi)* and *smarcc2(RNAi)* (adj. *p* <0.05, no fold cut-off) [115]. MA plots were made using ggplot R package and heatmaps were made by pheatmap R package. A spreadsheet containing aliases of the (NCBI Bio Project PRJNA215411) transcriptome to other planarian transcriptomes is provided (Additional file 12).

### Assay for transposase-accessible chromatin using sequencing (ATACseq)

Omni-ATAC-seq was performed with minor modifications to the published protocol [116, 117]; the precise methodology used is described as follows. The planarian stem cell (X1) fraction was isolated after F4D3 *control(RNAi)*, *brg1(RNAi)*, and *smarcc2l(RNAi)* by fluorescence activated cell sorting (FACS) as previously described [52, 88]. The isolated nuclei were incubated in the Tn5 transposase mix (Illumina, 20034197; 25μl 2× TD buffer, 2.5μl transposase (100nM final), 16.5μl PBS, 0.5μl 1% digitonin, 0.5μl 10% Tween-20, 5μl water) for 30 min in a thermomixer at 37°C with shaking at 1000 RPM to obtain genomic DNA enriched for accessible chromatin regions. Reactions were cleaned up with Zymo DNA Clean and Concentrator 5 columns (D4004).

Following purification, we amplified library fragments using the following PCR conditions: 72°C for 5 min, 98°C for 30 s, followed by thermocycling at 98°C for 10 s, 63°C for 30 s, and 72°C for 1 min for a maximum of 12 cycles. Cycle number was determined by amplifying a 5µL aliquot after 5 initial cycles for 20 additional cycles with Sybr Gold, and then determining the number of additional cycles required for the remaining 45µL reaction to reach 1/4 to 1/3 of the maximum fluorescence intensity [116]. Libraries were cleaned up with Zymo DNA Clean and Concentrator 5 columns (D4004). Libraries were size selected using PippinHT (Sage Science) for 100–600 bp. An Agilent Bioanalyzer was used to check library quality and concentration. All libraries were 50-bp paired end sequenced on an Illumina NovaSeq platform to a minimum depth of 36 million reads per library (Additional file 6). ATAC seq was performed in triplicate.

All ATACseq and RNAseq raw data were uploaded to NCBI Sequence Read Archives under BioProject PRJNA982893.

### ATACseq differential accessible (DA) peak analyses

Raw reads were preprocessed by Trimmomatic to remove adapters and low-quality reads [113] (LEADING:20 TRAILING:20 SLIDINGWINDOW:5:25 MINLEN:36) before being aligned to schMedS2 (NNSW00000000.1) planarian genome assembly [54]. The genome was indexed using bowtie2-build and fastq files were aligned to the genome using bowtie2 [118]. Reads mapped to the nonmitochondrial genome and with mapping quality scores > 30 were kept for downstream analysis using SAMtools (v 1.16.1) [119, 120] (samtools view -b -q 30). SAMtools was used to determine insert sizes—a successful ATACseq experiment should periodic fragment sizes correlating with nucleosome size. One library failed (control_1) and thus was excluded from further analysis (Additional file 13). Initial peak calling of each library were conducted by MACS2 (version 2.7.9) (--nolambda --nomodel --cutoff-analysis -B --keepdup all --gsize 7.6e8) [121]. Peaks showing enriched signals in one condition versus the other were identified using DiffBind package (version 3.0.15) [122] (DESeq2, FDR < 0.05, no fold cut-off). We used the output from DiffBind for all downstream analyses. The R package ChIPSeeker (version 1.26.2) [123, 124] was used to annotate peaks to the closest mapped transcript and generate pie chartdeepTools (version 3.5.0) [125] was used to generate normalized coverage files (bamCoverage –binSize 1 –normalizeUsing CPM) and WiggleTools [126] was used to generate average bigwig files for replicates of the same condition displayed in the genome browser tracks. IGV was used to display bigwig files (peak data) against genome tracks [127]. GMAP [128] was used to generate a gff file mapping the planarian Smed_ASXL transcriptome (NCBI Bio Project PRJNA215411) to the genome, which was displayed with SMESG high-confidence annotations [129]. Peak files were group-autoscaled for visualization purposes on IGV [127].

### HOMER de novo motif enrichment analysis in DA peaks

HOMER v4.11 [95] (http://homer.ucsd.edu/) was used to identify motifs enriched in differentially accessible regions following BAF knockdown. Specifically, DA peaks located within 1 kb from a TSS that were inaccessible in both *brg1* and *smarcc2* RNAi conditions were used as input for the findMotifsGenome.pl function with default parameters. De novo motif discovery results and statistics are presented in Additional file 11.

### Supplementary Information

**Additional file 1.** Planarian homologs for human BAF complex and corresponding RNAi phenotypes.

**Additional file 2.** Expression of planarian orthologs of human BAF complex subunits. (A)WISH of planarians homologs of human BAF complex subunits in WT planarians. (B) Single-cell RNA sequencing detection [57]. The transcriptome of a cell dictates its unique cell-type biology. We used published scRNAseq atlas data to determine the detection for each gene listed as well as the relative expression in bulk sequencing data from purified cell populations (bar graphs) [44, 57].

**Additional file 3.** The BAF complex is not required for cell viability but is required for differentiation. (.tif) (A) Whole worm TUNEL during the RNAi time course. TUNEL+ cells are quantified in the graph at the top right. (B) FISH for *piwi-1 *with immunostaining of anti-PIWI-1. Planarians were imaged with photoreceptors centered. The proportion of *piwi-1-*PIWI-1+ to *piwi-1+*PIWI-1+ cells are quantified below images. Quantifications are mean 1 S.D Significance levels in plots: *p<0.05,**p<0.01, ***p<0.001, each n is a representative region from 1 planarian.

**Additional file 4.** Differentially expressed transcripts in X2 cells during *brg1(RNAi) *and*smarcc2(RNAi) *versus *control(RNAi).*Sequencing metrics are included.

**Additional file 5.** Shared differentially expressed transcripts in X1s during *brg1(RNAi) *and*smarcc2(RNAi) *versus *control(RNAi). *Sequencing metrics are included.

**Additional file 6.** Shared differentially accessible peaks in *brg1(RNAi) *and *smarcc2(RNAi)*versus *control(RNAi). *Sequencing metrics are included.

**Additional file 7.** Chromatin accessibility and gene expression changes are correlated in planarian X1s. (A) FISH for pooled primer probe *soxP-1/soxP-2*and *piwi-1* marking s- neoblasts. Planarians were imaged in the boxed region indicated by the diagram on the left. *soxP-1/soxP-2+piwi-1+ *cells to total *piwi-1+ *cells are quantified in the graph to the right. (B) DA peaks associated by proximity to gene loci that cause changes in that transcript abundance. (C) Average change in transcript abundance based on location of DA peaks relative to gene loci of dysregulated genes with associated DA peaks. (D) DA peaks associated with unique transcripts based on proximity to gene loci compared with dysregulated transcripts. Quantifications are mean 1 S.D Significance levels in plots: *p<0.05,**p<0.01, ***p<0.001, each “n” is a representative region from 1 planarian.

**Additional file 8.** Accessibility and expression of cathepsin lineage-associated genes including *ets-1 *are disrupted in *brg1 *and *smarcc2 *RNAi. (A) ATAC-seq peaks identified in X1s during RNAi at *ets-1 *loci. The sequence coverage tracks show replicate-averaged, sequence depth-normalized (CPM) read coverage for each condition. (B) Heatmap of differentially expressed genes associated with the cathepsin lineage from RNAi conditions in X1s. Replicates are displayed independently. (C) FISH of *ets-1 *and immunostaining of PIWI-1 during RNAi at F4D3. Planarians were imaged in the boxed region to the left of the images. Quantifications of *ets-1+*PIWI-1+ to total PIWI-1+ cells are indicated in the graph to the right. Quantifications are mean 1 S.D Significance levels in plots: *p<0.05,**p<0.01, ***p<0.001, each “n” is a representative region from 1 planarian.

**Additional file 9.** Differential peaks and transcript expression of genes enriched in *tgs-1+*S/G2/M cells versus *tgs-1*- S/G2/M cells and nu-neoblasts.

**Additional file 10.** Progenitors of neural lineages are reduced in *brg1 *and *smarcc2* RNAi. (A) FISH of *ChAT *and immunostaining of PIWI-1 during RNAi at F4D3. Planarians were imaged in the boxed region to the left of the images. Quantifications of *ChAT+*PIWI-1+ to total PIWI-1+ cells are indicated in the graph to the right. (B) FISH of *transcript *indicated (green)and immunostaining of PIWI-1 (magenta) during RNAi at F4D3. Planarians were imaged in the boxed region to the left of the images. Quantifications of *transcript+*PIWI-1+ to total PIWI-1+ cells are indicated in the graph to the right. (C) FISH of *ovo*marking photoreceptors and photoreceptor progenitors during RNAi at F4D3. Planarians were imaged in the boxed region to the left of the images. *ovo*+ progenitors (*ovo+ *cells training posterior to photoreceptors) are quantified in the graph to the right. Quantifications are mean 1 S.D Significance levels in plots: *p<0.05,**p<0.01, ***p<0.001, each “n” is a representative region from 1 planarian.

**Additional file 11.** The summary of HOMER analysis on DA chromatin peaks controlled by both smarcc2 and brg1 within 1kb of a TSS.

**Additional file 12.** Alias list of Smed_ASXL transcripts to other common transcriptomes (NCBI Bio Project PRJNA215411). Listed is the top forward BLASTn result of a given Smed­_ASXL transcript to the entire other transcriptome.

**Additional file 13.** Quality control assessment of ATAC sequencing. Distribution of insert sizes from ATAC sequencing of all replicates to ensure successful Tn5 transposase reaction and quality sequencing. Control_x1_2 was excluded from further analysis, as lack of periodicity of insert sizes (due to nucleosome placement) indicates failed transposition.

## Data Availability

All raw data are publicly available. Materials can be provided upon request. The RNA-seq and ATAC-seq data from this study have been deposited to the NCBI Gene Expression Omnibus (BioProject accession number PRJNA982893 [130].

## Abbreviations

ASC: Adult stem cell
BAF: Brahma-Associated Factor
BRG: Brahma Related Gene
scRNAseq: Single-cell RNA sequencing
ATACseq: Assay of Transposase Accessible Chromatin sequencing
RNAi: RNA interference
DA: Differentially accessible
DE: Differentially expressed
TSS: Transcriptional start site
UTR: Untranslated region
WISH: Whole mount in situ hybridization
FISH: Fluorescent in situ hybridization
WT: Wild type
PCG: Positional control gene
TF: Transcription factor
H3P: Histone H3 phosphorylated on serine 10
TUNEL: Terminal deoxynucleotidyl transferase dUTP Nick End Labeling
GFP: Green fluorescent protein
FACS: Fluorescence activated cell sorting
