# Supplementary figures and images for: The BAF chromatin remodeling complex licenses planarian stem cells access to ectodermal and mesodermal cell fates
Source: BMC Biol. 2023 Oct 20;21:227. doi: 10.1186/s12915-023-01730-y (PMC10589948; doi:10.1186/s12915-023-01730-y)

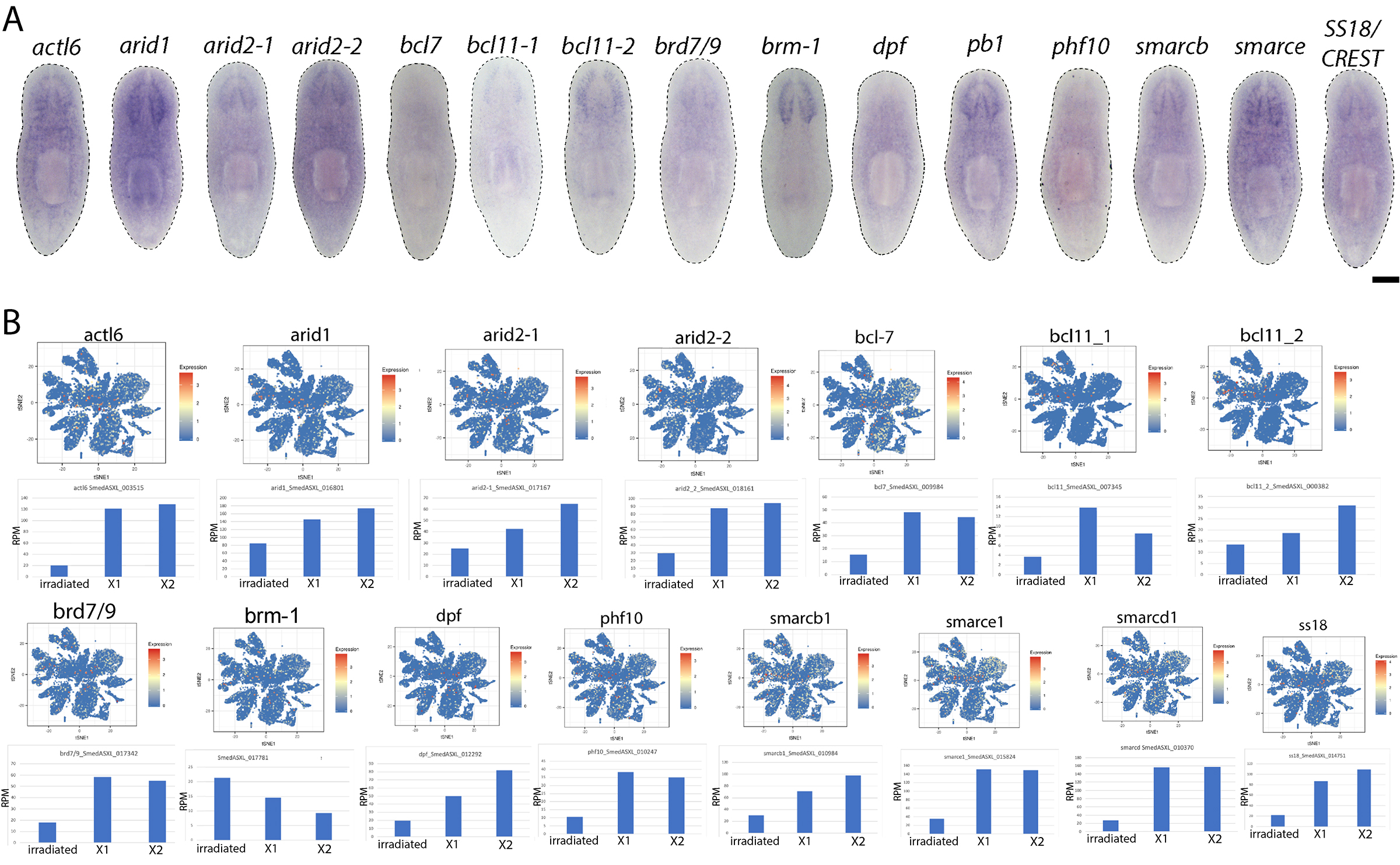

Supplement: Supplementary file 2 — Additional file 2. Expression of planarian orthologs of human BAF complex subunits. (A)WISH of planarians homologs of human BAF complex subunits in WT planarians. (B) Single-cell RNA sequencing detection [57]. The transcriptome of a cell dictates its unique cell-type biology. We used published scRNAseq atlas data to determine the detection for each gene listed as well as the relative expression in bulk sequencing data from purified cell populations (bar graphs) [44, 57]. [file 12915_2023_1730_MOESM2_ESM.jpg]

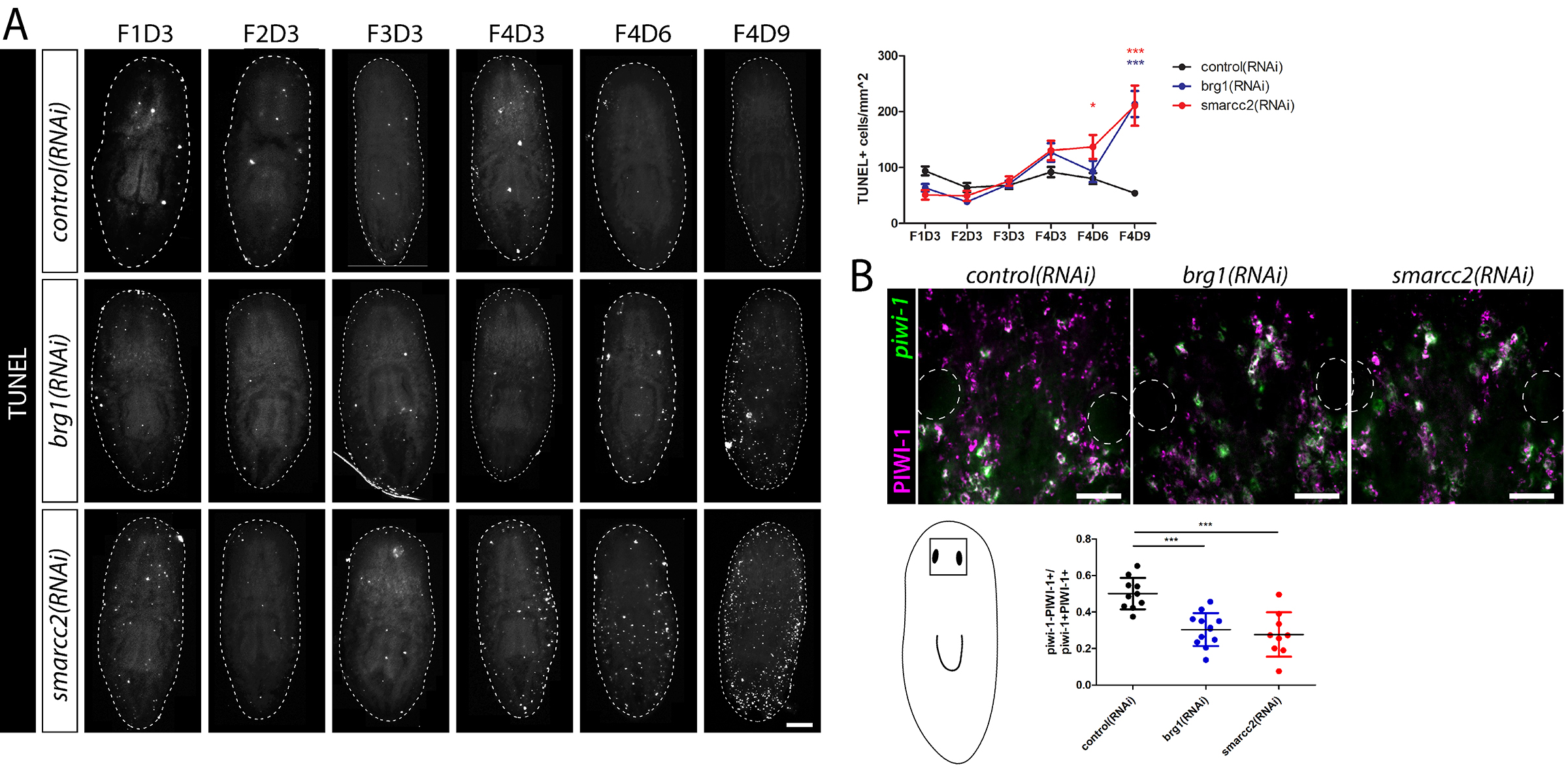

Supplement: Supplementary file 3 — Additional file 3. The BAF complex is not required for cell viability but is required for differentiation. (.tif) (A) Whole worm TUNEL during the RNAi time course. TUNEL+ cells are quantified in the graph at the top right. (B) FISH for piwi-1 with immunostaining of anti-PIWI-1. Planarians were imaged with photoreceptors centered. The proportion of piwi-1-PIWI-1+ to piwi-1+PIWI-1+ cells are quantified below images. Quantifications are mean 1 S.D Significance levels in plots: *p<0.05,**p<0.01, ***p<0.001, each n is a representative region from 1 planarian. [file 12915_2023_1730_MOESM3_ESM.jpg]

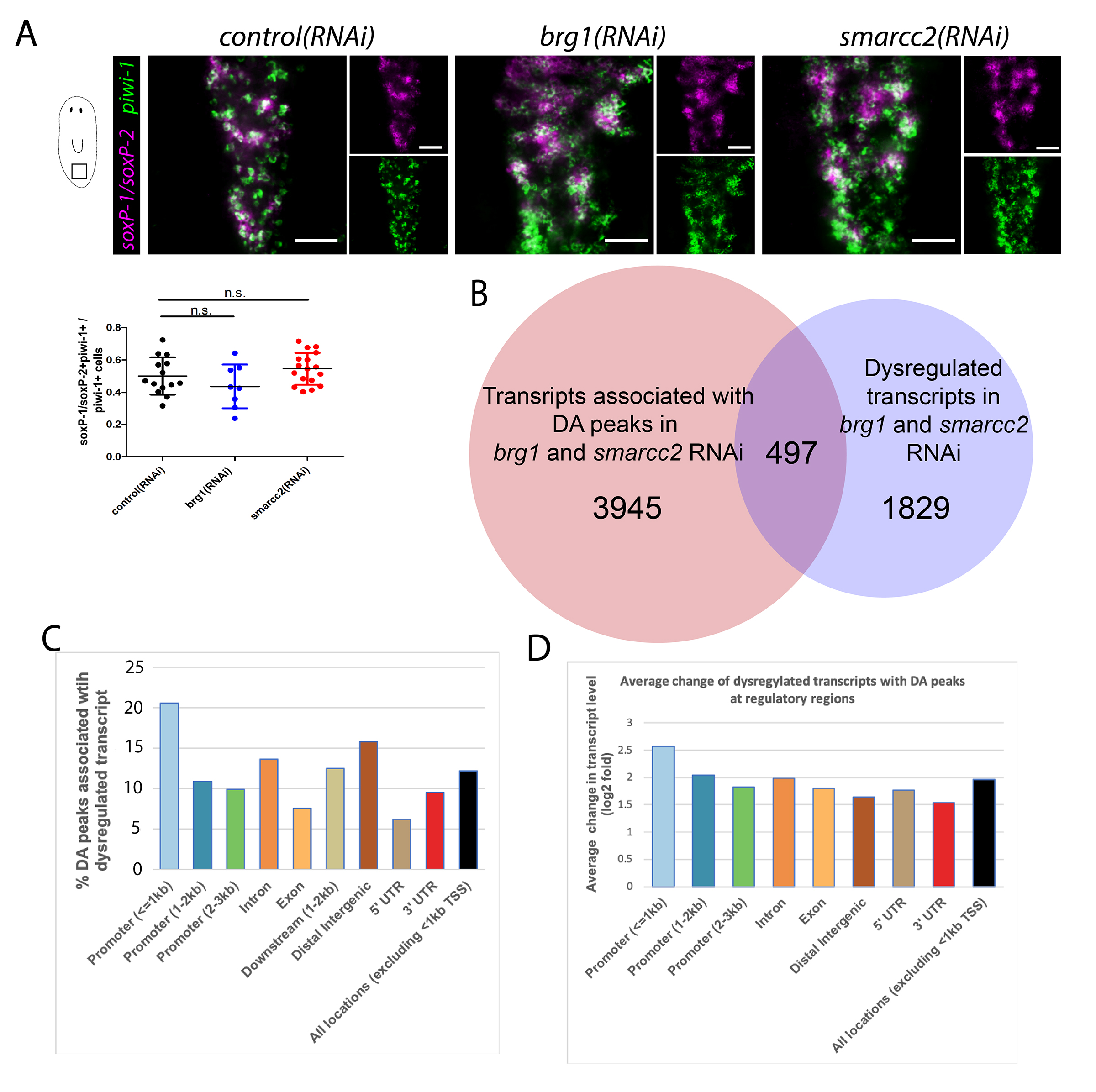

Supplement: Supplementary file 7 — Additional file 7. Chromatin accessibility and gene expression changes are correlated in planarian X1s. (A) FISH for pooled primer probe soxP-1/soxP-2and piwi-1 marking s- neoblasts. Planarians were imaged in the boxed region indicated by the diagram on the left. soxP-1/soxP-2+piwi-1+ cells to total piwi-1+ cells are quantified in the graph to the right. (B) DA peaks associated by proximity to gene loci that cause changes in that transcript abundance. (C) Average change in transcript abundance based on location of DA peaks relative to gene loci of dysregulated genes with associated DA peaks. (D) DA peaks associated with unique transcripts based on proximity to gene loci compared with dysregulated transcripts. Quantifications are mean 1 S.D Significance levels in plots: *p<0.05,**p<0.01, ***p<0.001, each “n” is a representative region from 1 planarian. [file 12915_2023_1730_MOESM7_ESM.jpg]

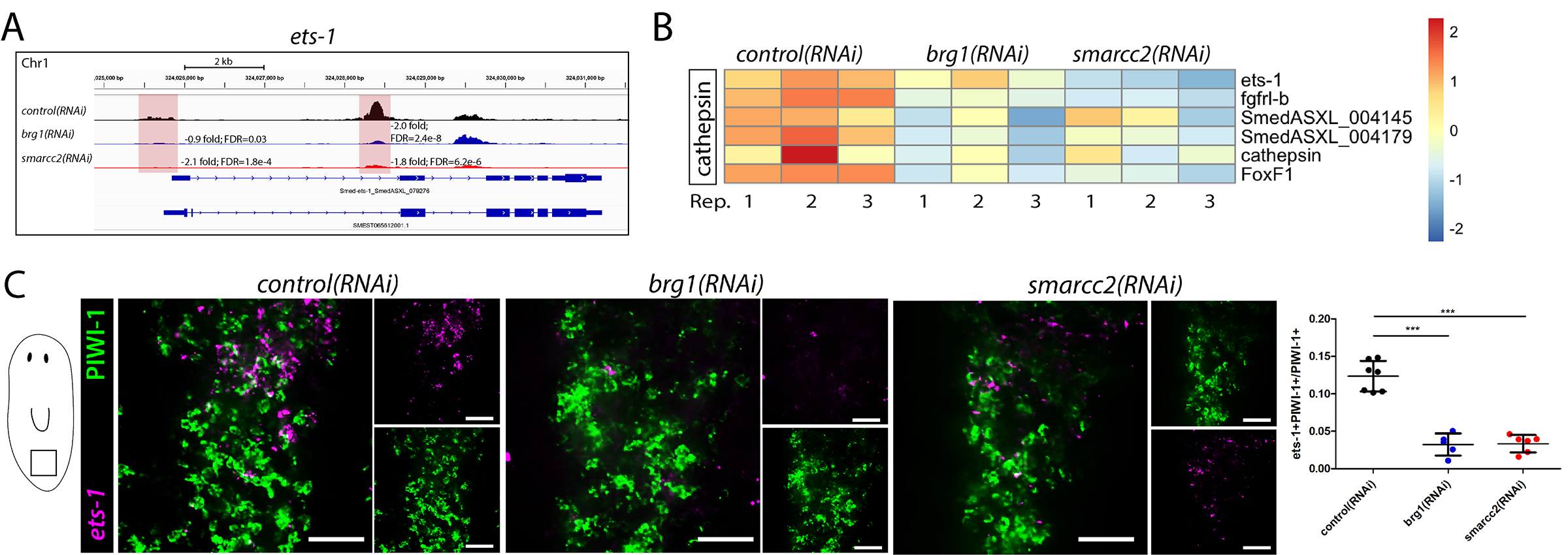

Supplement: Supplementary file 8 — Additional file 8. Accessibility and expression of cathepsin lineage-associated genes including ets-1 are disrupted in brg1 and smarcc2 RNAi. (A) ATAC-seq peaks identified in X1s during RNAi at ets-1 loci. The sequence coverage tracks show replicate-averaged, sequence depth-normalized (CPM) read coverage for each condition. (B) Heatmap of differentially expressed genes associated with the cathepsin lineage from RNAi conditions in X1s. Replicates are displayed independently. (C) FISH of ets-1 and immunostaining of PIWI-1 during RNAi at F4D3. Planarians were imaged in the boxed region to the left of the images. Quantifications of ets-1+PIWI-1+ to total PIWI-1+ cells are indicated in the graph to the right. Quantifications are mean 1 S.D Significance levels in plots: *p<0.05,**p<0.01, ***p<0.001, each “n” is a representative region from 1 planarian. [file 12915_2023_1730_MOESM8_ESM.jpg]

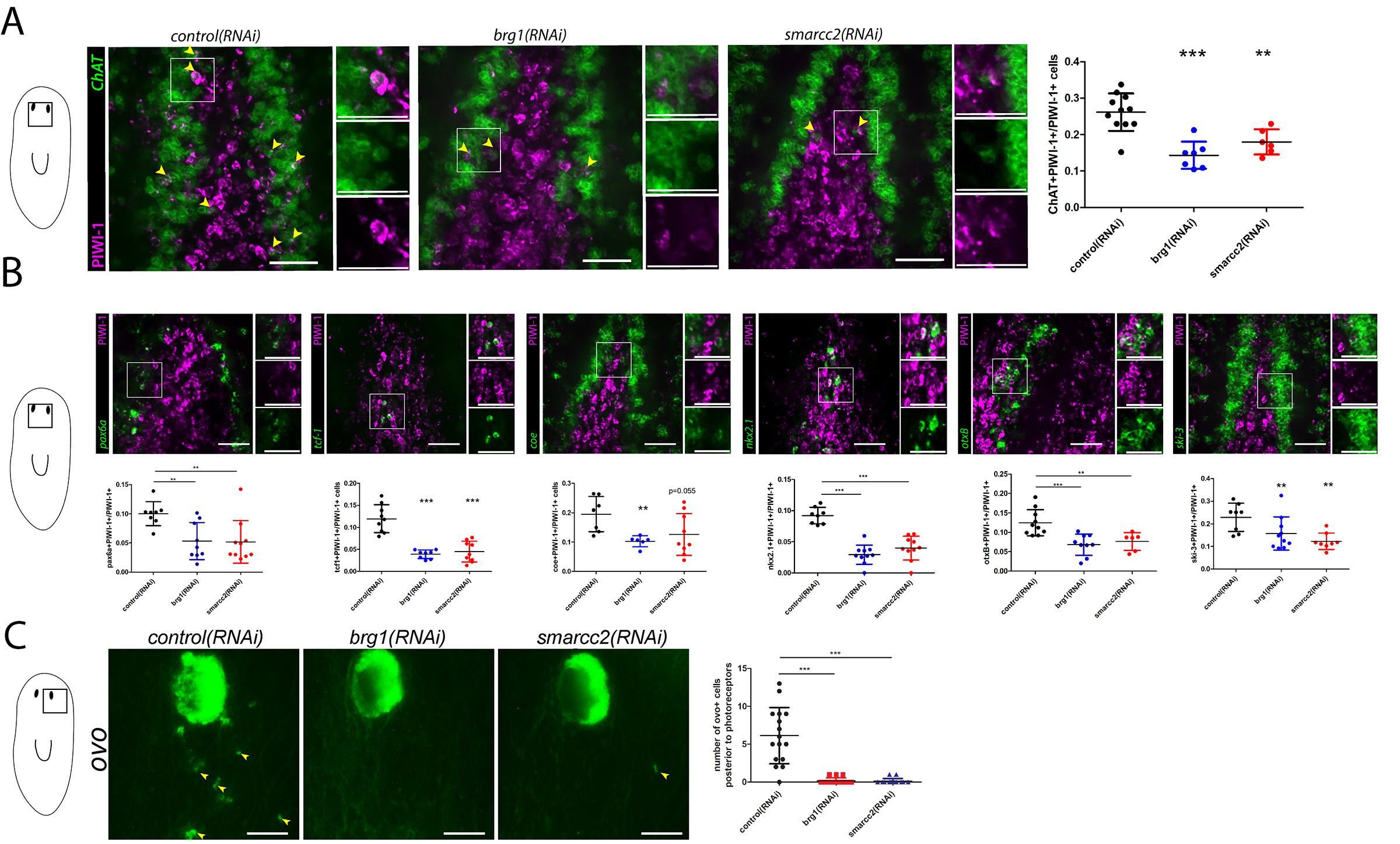

Supplement: Supplementary file 10 — Additional file 10. Progenitors of neural lineages are reduced in brg1 and smarcc2 RNAi. (A) FISH of ChAT and immunostaining of PIWI-1 during RNAi at F4D3. Planarians were imaged in the boxed region to the left of the images. Quantifications of ChAT+PIWI-1+ to total PIWI-1+ cells are indicated in the graph to the right. (B) FISH of transcript indicated (green)and immunostaining of PIWI-1 (magenta) during RNAi at F4D3. Planarians were imaged in the boxed region to the left of the images. Quantifications of transcript+PIWI-1+ to total PIWI-1+ cells are indicated in the graph to the right. (C) FISH of ovomarking photoreceptors and photoreceptor progenitors during RNAi at F4D3. Planarians were imaged in the boxed region to the left of the images. ovo+ progenitors (ovo+ cells training posterior to photoreceptors) are quantified in the graph to the right. Quantifications are mean 1 S.D Significance levels in plots: *p<0.05,**p<0.01, ***p<0.001, each “n” is a representative region from 1 planarian. [file 12915_2023_1730_MOESM10_ESM.jpg]

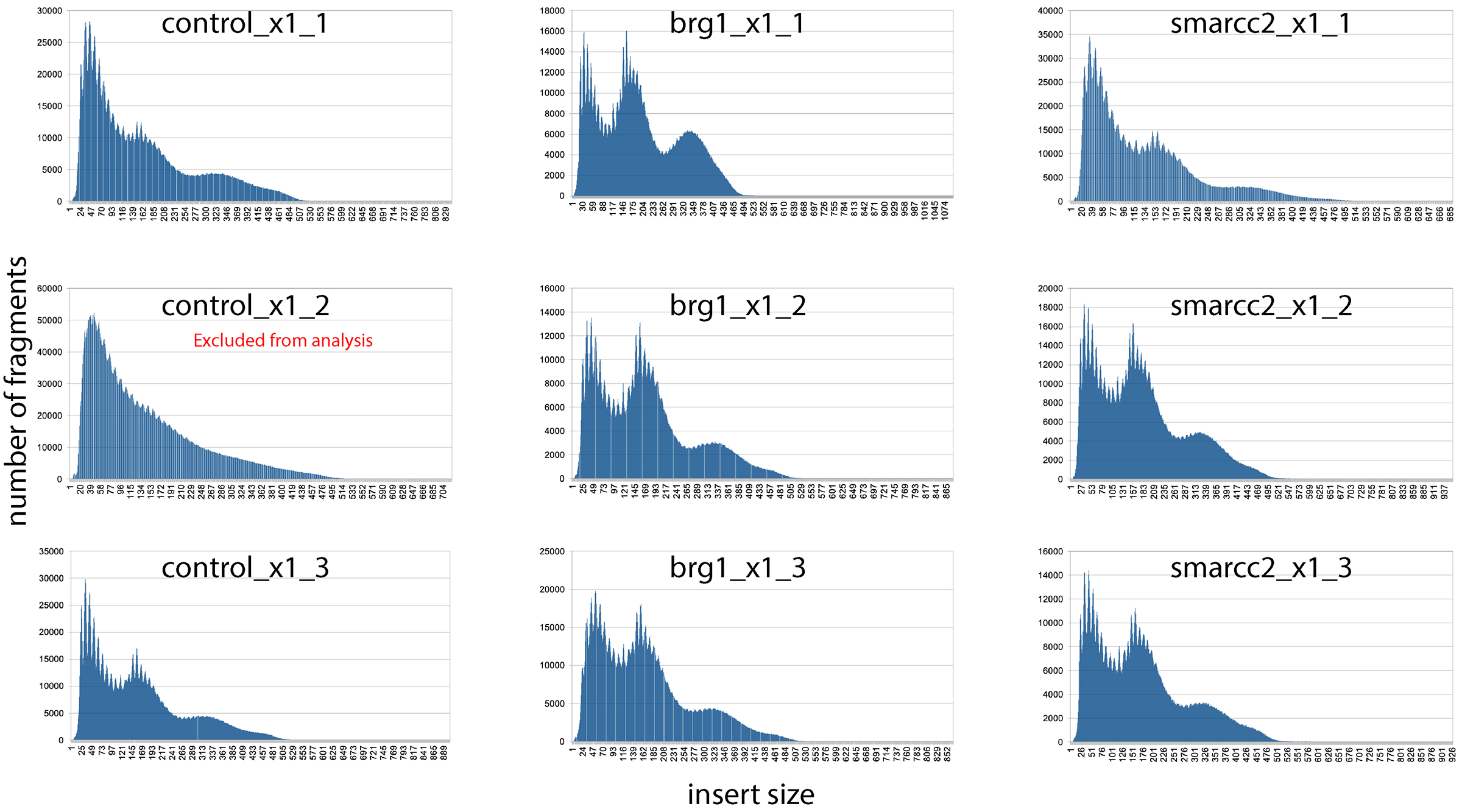

Supplement: Supplementary file 13 — Additional file 13. Quality control assessment of ATAC sequencing. Distribution of insert sizes from ATAC sequencing of all replicates to ensure successful Tn5 transposase reaction and quality sequencing. Control_x1_2 was excluded from further analysis, as lack of periodicity of insert sizes (due to nucleosome placement) indicates failed transposition. [file 12915_2023_1730_MOESM13_ESM.jpg]
