# Supplementary material for: The BAF chromatin remodeling complex licenses planarian stem cells access to ectodermal and mesodermal cell fates
Source: BMC Biol. 2023 Oct 20;21:227. doi: 10.1186/s12915-023-01730-y (PMC10589948; doi:10.1186/s12915-023-01730-y)
Supplement: Supplementary file 11 — Additional file 11. The summary of HOMER analysis on DA chromatin peaks controlled by both smarcc2 and brg1 within 1kb of a TSS. [file 12915_2023_1730_MOESM11_ESM.docx]

| **Motif logo** | **Motif name** | **p-value** | **q-value (Benjamini)** | **% of Target Sequences with Motif** | **% of Background Sequences with Motif** | **Role in planarians** |
| --- | --- | --- | --- | --- | --- | --- |
| 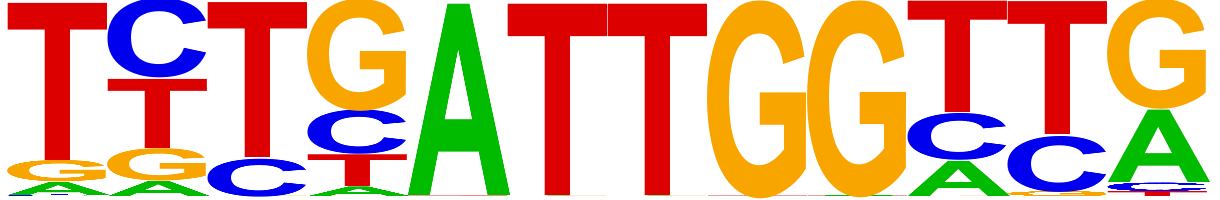 | HAP3 | 1e-235 | -5.432e+02 | 21.39 | 2.85 | Promotes self-renewal and proliferation of early germ cells ^1^ |
| 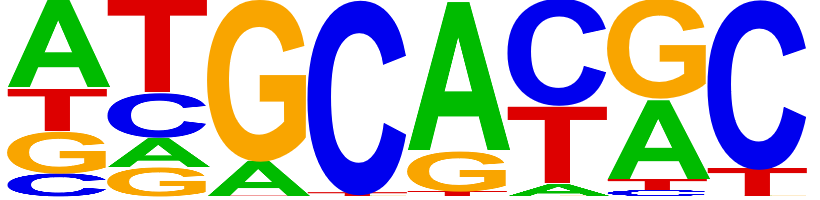 | RBFox2 | 1e-147 | -3.387e+02 | 41.71 | 17.23 |  |
| 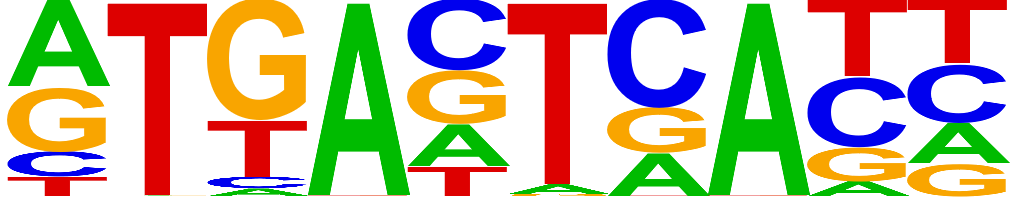 | Fra1 | 1e-83 | -1.917e+02 | 26.79 | 11.20 |  |
| 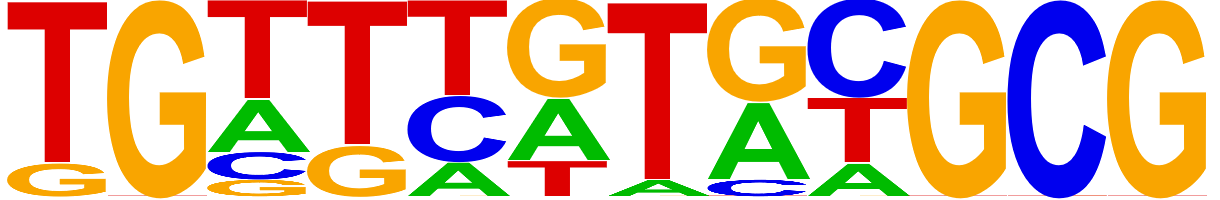 | FUS | 1e-77 | -1.790e+02 | 4.91 | 0.34 |  |
| 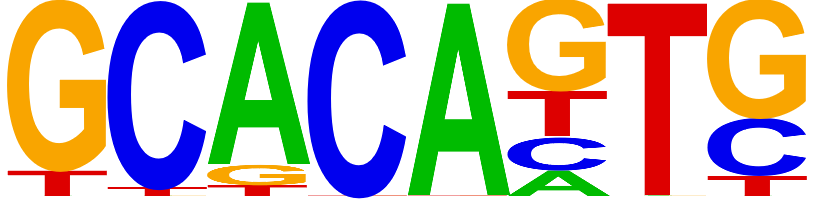 | MEC-8 | 1e-45 | -1.037e+02 | 31.50 | 18.43 |  |
| 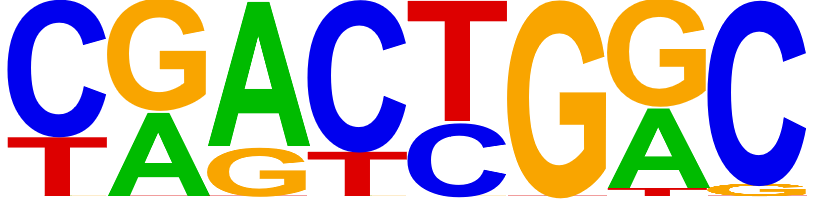 | Initiator | 1e-38 | -8.924e+01 | 16.14 | 7.44 |  |
| 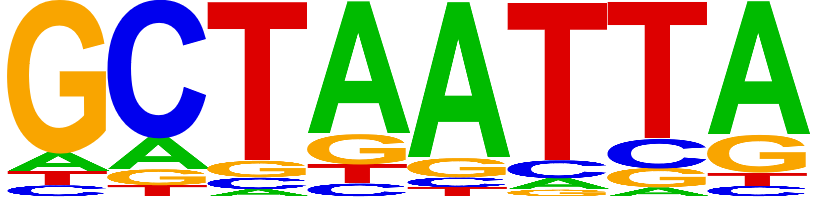 | Ro | 1e-31 | -7.329e+01 | 57.61 | 44.52 |  |
| 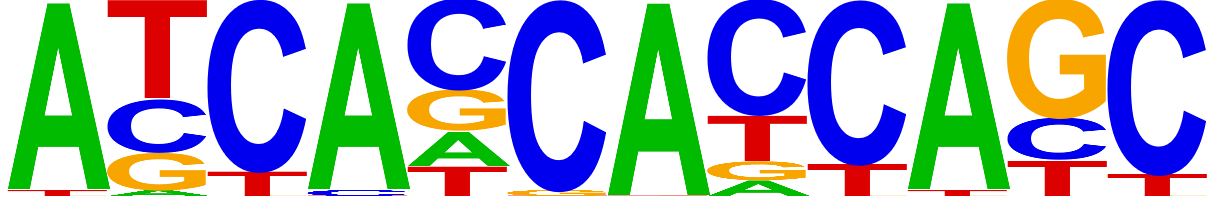 | ZML2 | 1e-30 | -6.917e+01 | 3.58 | 0.64 |  |
| 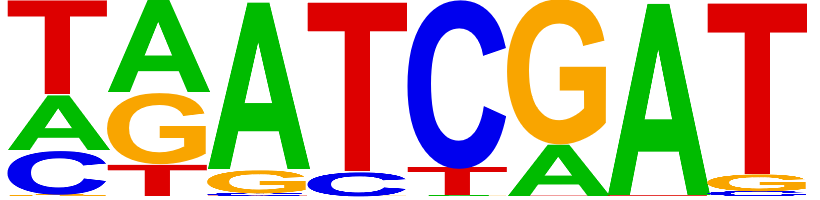 | CUX1 | 1e-29 | -6.875e+01 | 30.81 | 20.08 | Putative marker of planarian neural progenitors ^2^ |
| 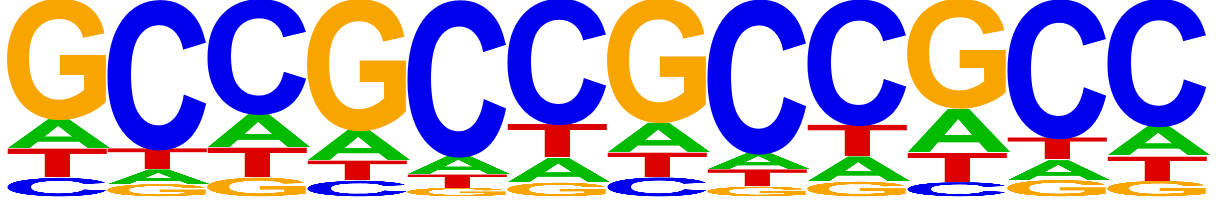 | CRF4 | 1e-27 | -6.356e+01 | 7.31 | 2.60 |  |
| 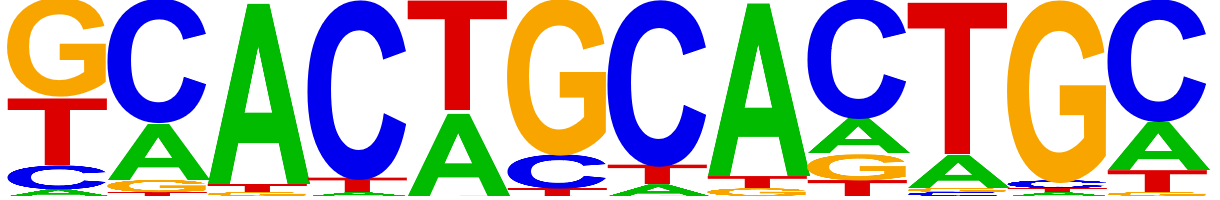 | PB0091.1_Zbtb3_1 | 1e-20 | -4.626e+01 | 5.15 | 1.80 |  |
| 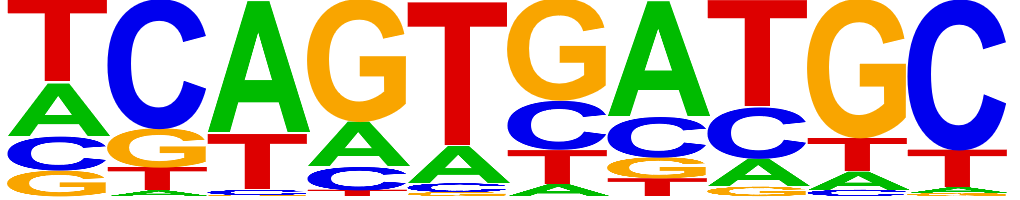 | PAX6 | 1e-18 | -4.360e+01 | 2.75 | 0.62 | Expressed broadly in the brain, eyes, and ventral nerve cords and in a subset of neoblasts ^3,4^ |
| 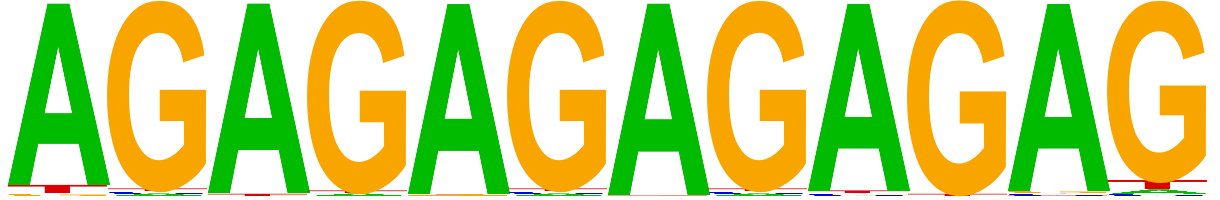 | RAMOSA1 | 1e-18 | -4.235e+01 | 1.32 | 0.12 |  |
| 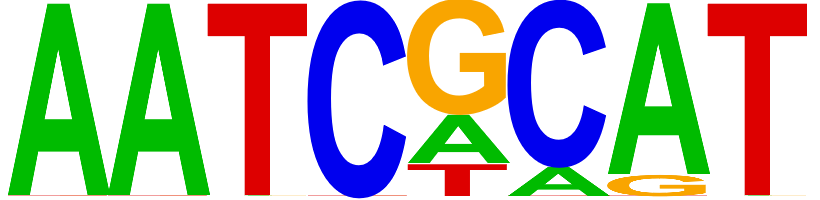 | Prop1 | 1e-17 | -4.136e+01 | 9.96 | 5.15 |  |
| 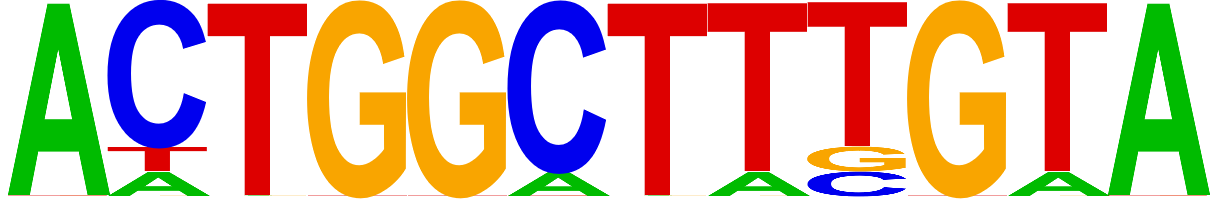 | grh | 1e-16 | -3.804e+01 | 0.49 | 0.01 |  |
| 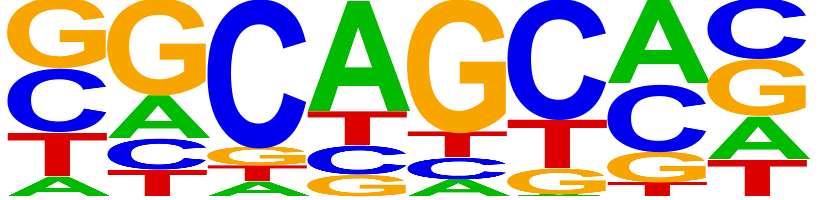 | SWI5 | 1e-15 | -3.542e+01 | 6.43 | 2.94 |  |
| 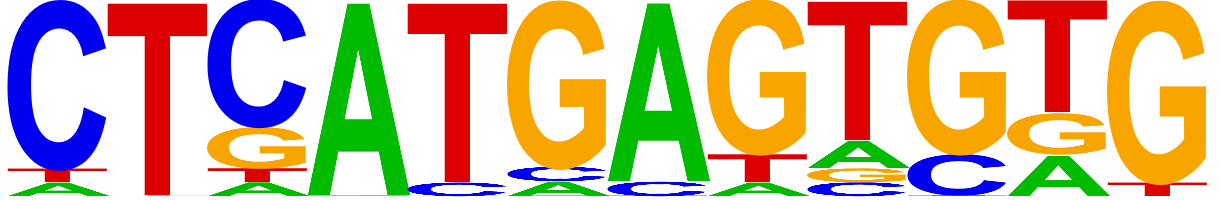 | z | 1e-14 | -3.381e+01 | 0.79 | 0.04 |  |
| 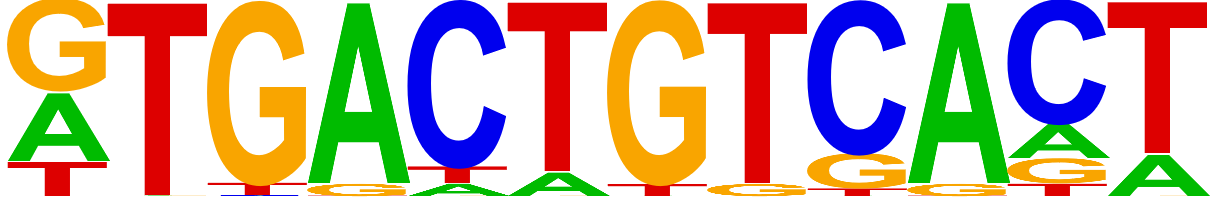 | SIZF2 | 1e-13 | -3.135e+01 | 0.79 | 0.05 |  |
| 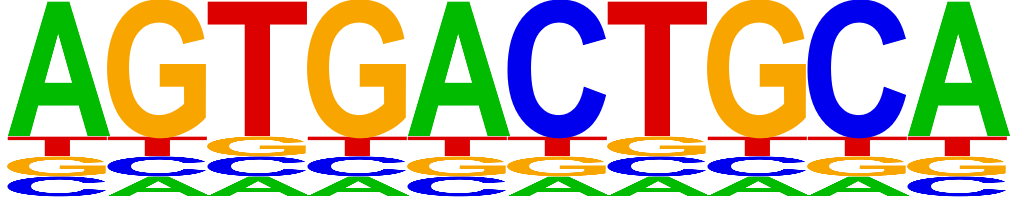 | STZ | 1e-13 | -3.038e+01 | 21.00 | 14.85 |  |
| 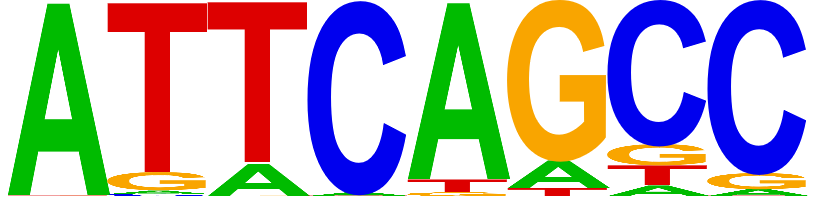 | SWI5 | 1e-12 | -2.940e+01 | 8.00 | 4.33 |  |
| 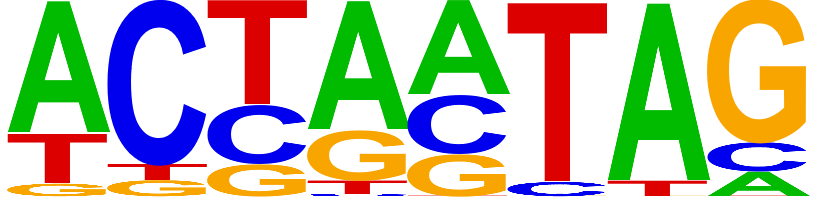 | br-Z2 | 1e-12 | -2.894e+01 | 5.50 | 2.58 |  |

**References:**

1. Iyer, H., Collins, J. J. & Newmark, P. A. NF-YB Regulates Spermatogonial Stem Cell Self-Renewal and Proliferation in the Planarian Schmidtea mediterranea. *PLoS Genet* **12**, e1006109 (2016).

2. Molinaro, A. M. & Pearson, B. J. In silico lineage tracing through single cell transcriptomics identifies a neural stem cell population in planarians. *Genome Biol* **17**, (2016).

3. Scimone, M. L. L., Kravarik, K. M. M., Lapan, S. W. W. & Reddien, P. W. W. Neoblast specialization in regeneration of the planarian Schmidtea mediterranea. *Stem Cell Reports* **3**, 339–352 (2014).

4. Pineda, D. *et al.* The genetic network of prototypic planarian eye regeneration is Pax6 independent. *Development* **129**, 1423–1434 (2002).
